# Supplementary material for: A neuropeptide modulates sensory perception in the entomopathogenic nematode Steinernema carpocapsae
Source: PLoS Pathog. 2017 Mar 2;13(3):e1006185. doi: 10.1371/journal.ppat.1006185 (PMC5333901; doi:10.1371/journal.ppat.1006185)
Supplement: S1 Table — (DOCX) [file ppat.1006185.s001.docx]

**Table S1. *Steinernema carpocapsae* argonaute proteins.**

| AGOs Protein List | Homologues | Accession * |
| --- | --- | --- |
| ALG-1 | x2 | L596_g7718.t1 |
| ALG-2 | x1 | L596_g16709.t1 |
| ALG-3 (T22B3.2) | x2 | L596_g3457.t1 |
| ALG-4 |  |  |
| CSR-1 | x1 | L596_g20174.t1 |
| C04F12.1 | x1 | L596_g11107.t1 |
| C14B1.7a |  |  |
| ERGO-1 |  |  |
| HPO-24 |  |  |
| HRDE-1 (WAGO-9) | x1 | L596_g11197.t1 |
| NRDE-3 (WAGO-12) |  |  |
| PPW-1 (WAGO-7) |  |  |
| PPW-2 (WAGO-3) |  |  |
| PRG-1 | x1 | L596_g25491.t1 |
| RDE-1 |  |  |
| SAGO-2 (WAGO-6) |  |  |
| SAGO-1 (WAGO-8) |  |  |
| T23B3.2 | x1 | L596_g21112.t1 |
| WAGO-1 (R06C7.1) | x19 | L596_g19943.t1 |
| WAGO-10 (T22H9.3) | x2 | L596_g19923.t1 |
| WAGO-11 (Y49F6A.1) | x3 | L596_g17524.t1 |
| WAGO-2 (F55A12.1) | x1 | L596_g16917.t1 |
| WAGO-4 (F58G1.1) |  |  |
| WAGO-5 (ZK1248.7) | x4 | L596_g12936.t1 |

* Accession numbers presented are the top returning hits for each RNAi pathway protein.
