# Supplementary material for: A neuropeptide modulates sensory perception in the entomopathogenic nematode Steinernema carpocapsae
Source: PLoS Pathog. 2017 Mar 2;13(3):e1006185. doi: 10.1371/journal.ppat.1006185 (PMC5333901; doi:10.1371/journal.ppat.1006185)
Supplement: S2 Table — (DOCX) [file ppat.1006185.s002.docx]

**Table S2. *Steinernema carpocapsae* non-argonaute RNA interference pathway proteins.**

| Non AGO Protein List | Homologues | Accession * |
| --- | --- | --- |
| AIN-1 | x1 | L596_g5788.t1 |
| DCR-1 | x1 | L596_g26648.t1 |
| DRH-1 | x1 | L596_g26385.t1 |
| DRH-3 | x1 | L596_g25860.t1 |
| DRSH-1 | x4 | L596_g13580.t1 |
| EGO-1 | X3 | L596_g919.t1 |
| EKL-1 |  |  |
| ERI-1 | x5 | L596_g16241.t2 |
| ERI-3 |  |  |
| ERI-5 |  |  |
| FKH-3 |  |  |
| FKH-4 |  |  |
| FKH-5 | x1 | L596_g10448.t1 |
| PASH-1 | x2 | L596_g24740.t1 |
| RDE-4 |  |  |
| RRF-1 | x1 | L596_g7841.t1 |
| RRF-3 | x1 | L596_g11915.t1 |
| RSD-3 | x1 | L596_g5786.t2 |
| RSD-6 |  |  |
| SID-1 |  |  |
| SID-2 |  |  |
| TSN-1 | x1 | L596_g28061.t1 |
| UNC-130 | x1 | L596_g11813.t1 |
| VIG-1 | x1 | L596_g22586.t1 |
| XPO-5 |  |  |
| XRN-1 | x1 | L596_g18210.t1 |
| XRN-2 | x1 | L596_g10155.t1 |
