# Supplementary material for: A neuropeptide modulates sensory perception in the entomopathogenic nematode Steinernema carpocapsae
Source: PLoS Pathog. 2017 Mar 2;13(3):e1006185. doi: 10.1371/journal.ppat.1006185 (PMC5333901; doi:10.1371/journal.ppat.1006185)
Supplement: S1 Text — (DOCX) [file ppat.1006185.s003.docx]

**Figure S3. Alignment of *Sc-flp-21* dsRNA against most similar non-target *S. carpocapsae* gene (McWilliam et al., 2013).**

CLUSTAL O(1.2.1) multiple sequence alignment

exon_14_L596g5821 CAAATTCAAGAGCGCAGCAGGGATAAAGGCTTGTAGCTGGCCGCAAAAGCCATCAGACGC

*Sc-flp-21_dsRNA* ------------------------------------------------------------

exon_14_L596g5821 TAATTGCGCCAGAACTCGGTCAGCGTAGTATTCGGCGATCTTGCAAATGCATTGGACAAG

*Sc-flp-21_dsRNA* ------------------------------------------------------------

exon_14_L596g5821 TTTCTCCGTCAGGTCAGCCCTGAGAACTGCATCGCTGACATGCATCCTTTCCCAGATGGC

*Sc-flp-21_dsRNA* ------------------------------------------------------------

exon_14_L596g5821 CAAGTATTGATCGATGATGTGGCAAACATCTACATAAGAACTAGTGTGCCGAATCTCCGA

*Sc-flp-21_dsRNA* -------------------------------------------------------TTCTG

* * .

exon_14_L596g5821 AGACGCGATTTGGATTGTGGCGTCGAGTTGGCAAGCCAGTTCCACGCGCCCGAACG--CC

*Sc-flp-21_dsRNA* AGCCGCT---------------------ATCTGAGCCAGTTCAACGCGCCCGAATACGAC

**.*** : .*********.*********** . .*

exon_14_L596g5821 TTCAGCTTCGCCGTGTCCATCCA---CTTCACG--------ACGACTCGGTCGAAAATCT

*Sc-flp-21_dsRNA* CCCAGCGGCTACATGTACTTCGATCAGCGCTCGATGAAGCGAGGCCTCGGTCCTC-----

**** * .*.***.*:** * *:** * *.******* :.

exon_14_L596g5821 GGCTCCAGTCGGATTTGTCCAGCCGGTATTTCTGCTTTGGAAGACGGTACTGACGAAATT

*Sc-flp-21_dsRNA* ------------------------GACCTCTCCGCTTTGGTTAACTGCTA-----GAAAT

*. .* ** *******::.** * :. .**:*

exon_14_L596g5821 CTTGCGCTGCCAGATGTACCTTCAGAATCGTGATCAAAAGTTCTTCATTGTCGCCTTCGC

*Sc-flp-21_dsRNA* CGTGATATTTCAGAATTCTC------------CTCTTTTGATCTCTTTGATTGTTTGTTC

* **. .* ****: *. * .**::::*:*** :* .* * * *

exon_14_L596g5821 TCTTCGACGCGCTCATCAT

*Sc-flp-21_dsRNA* CCTGCGACT----------

** ****
