# Supplementary material for: A neuropeptide modulates sensory perception in the entomopathogenic nematode Steinernema carpocapsae
Source: PLoS Pathog. 2017 Mar 2;13(3):e1006185. doi: 10.1371/journal.ppat.1006185 (PMC5333901; doi:10.1371/journal.ppat.1006185)
Supplement: S2 Text — (DOCX) [file ppat.1006185.s004.docx]

RNAi pathway proteins

>AIN-1 - L596_g5788.t1

MWGEETGAETMTLNPWAGVSQQPQVWQNMNSRGNGNPSVANSVGSFAPNGGSVRPGSQQW

DGGATPAGRSAWNPNPHAPRWTPSVNHGNASGWPNAQQHWSVANSNGTYAEQTKKNMALG

RSSGPNLNMSQRFDGSQGWGSKVDQGTPWDTGNSGGVSQQMHEGKEQWSNPTRWTQPPGT

WPTPIVAPMVADWQQGQPRHDHWANHPTGGMVPSGGAWAQQALGVASSSEQGRVPYDPNP

QTPGPWVAPQPAEMNNDMMWHDPNPKQKKIQKDVGTGIWGDPTSQIEIRRWKDLEAEGGE

FPGGSDWGSGSNSSTQPTGWGDVGPAQGNDGTDRWGAQGQALQGGWSDKVDQDRDLNGGK

SDLNQIQRGIQNAIANGALCPDGQSRLQQWKKQRGGIGVGSPEEKMGVASSSDSNVPSLI

AATKIMNIHGDWTPPSSVADDSAKSEDQVSSSGTNNEKESSVKESASPTPPPTQLDDGPQ

EFVPGKKWEWRDPNKVAEDPNATPGNCKPNPLMAAGNNMNFAFSNSAVNTANPYGPEVPS

GNASTFWPNNSQGFNVPYGRDMYNSVRARLPSNGQFMPQRGMSGGYSNSNHNRMQTPPGK

GVFIVLNHQGANETQLNFSCTRAGQLLNVASLGGQTVLLRYADSSSEGVLQKLKADFPGN

ERIKTVSEEEVEKLLKNRSPTMSGSAWAPNSDTPLWSNGGSPLATGEIMSSQNVFSNQED

LNRQQY

>DCR-1 - L596_g26648.t1

MSKFAGKPPQAPPRPQKLCGLKSTTRRRGLHFDGRAPAAKSRFQISLANFKFVWRRSGDS

LADRLQKRDENWILKLRSPSDSAKAKNAAQMVVKATEINPNFFTPRDYQIELLEKALNYN

TIIPLGTGSGKTFIAVLLIKEYTQLLLPKFSNGGKRAVFIVDKVALVKQQADHIECHTEL

KVAQFHGYMNTDVCNTREAFEKVIEDSQVLVLTAQIFLMLLDHAIFSFDRVPVIVMDECH

HVLGGKHPYRLIVQRYSELSEETRPRMLGLTASLINNKTAPSELEALLRQLETIMKCRIE

ASSDIISVAKYGARPKELVIACRDNDFAEDWIRVCLKSLQNLRESAANCLDFHPDLDVDP

RKSVVEAATKILSVLQQMGPWCAWKVAMVWEKQLRKIHTSKSKASGLGEKQIAFLQNGES

KIHEIIKEFEQKVKTVRSYEELKKYIPHKVERLLELLRYYHPNIQKTLQGGGVKSLSAMV

FVDQRYVAYAMNLLMKSLYKWNQIDFGHLRSDFVIGFNGTSLGEESAGFHKRQETVLEKF

RQSQLNLLFTTSVLEEGVDVKHCNLVIKFDSPGDFRSYIQSRGRARKQGAHYFMLTEAKN

KLEFALNLRNFCEIERMLITRTVTIHDPEHQVPARNVDDVVQPYVVTSTGAKVTMSTAIA

LINKYCAKLPSDVFTRLVPQSCIEPVTVNGVTKFLATLSLPINSPFKEEIRLKTPMDTKK

LAQMAVALEACRQLHEKNELNDYLLPAGKDAIAATFLLDEDPDEYVPHMPHKAGSIRRKQ

LYDKKMDLLVEASGAANPKRRKIANPLDNDYFFGFLSKRILPEVPSFPIFPRQGKVLVSI

KLCKNQITLDEETFERAMKFHEYLFDDVLGLAKQGFVEFVPRHAPIATVIVPLKRREHAV

YDLDRVYLSNILEYRRIPYTPSEEERRTFQFREEDFTDAIVVPWYRNQDVFYDVAVINHS

LTPASGFPDGNYTNFREYYMKRYNLEIFNETQPLLDVDYTSTRMNLVMPRIPTRSQGKEK

KSDRTQHQVMVPELVNVHPIPASLWNSIVTLPTIIYRANALLLADELRELICAEALGPND

NSVHICWEPLDYVTSYSEDAQLPINKLSHLEKDLEAERAKARAENPEPEIVDMEDDSSND

GFNIGVWDPKLGDGGELAGRILTVPFPMASSARTDEEIADEAADMIVTSGNLNNGNMSDD

EDDQGEMKMLMDYAHIYKNDSSSFLPVRDNIEELGWDADIGQLNISEDAFPVSITGTSAS

IDGKSLMKDIASVLDPSFAGNGVASANGSAPNGSGDAVFAKSKPVATRLDLSAFDDRDAP

LIRTGENESLNLIEYFMDSNDSQEVERMKKASLEIESSRPHLENFPVVEVEEEGTPQKAI

EIDNSLFTNPNVLQPEINAYTDGDVGERSEVARVIPKTTFMKPDVKVNFTDEGMPECSAG

VSPCVLLQALTLSHASDGINLERLETVGDSFLKFAVTDYLFHEHKEQHEGKLSFARSKEV

SNYNLYRIGKKKNLPSILVASKFEPTDSWLPPCYMPTGDFKGPNAEDAEATDKFMDAVFE

GKPIPQSKKPLTGWDEDNNEAEKVVDGIETINLLKNPTGNTQYDVNEEISPLPYNVMTQQ

YISDKAIADTVEALIGAHLLQLGPKVALRFMRWLGLRVISEQTVADEPLLRFLDTPEDPN

RSNQELVSYIERFQLSSVEKTIGYRFNNKAYLLQAFTHASYYKNRITSCYQRLEFLGDAV

LDYMITRFLFQHRKKYSPGVLTDLRSALVNNTIFASLAVKYNFHKHFVAMCPGLHHMIEK

FVRLCESQAKNTNFNSELYMVTEEEFDDGDEEDIEVPKALGDIFESLAGAIYLDSGRRLD

VVWEVFYNLMHDTIMECCEHPPKSPIRELLEREPDKARFSKLERIRENGKIRVTVDIQGK

CRFTGIGRSYRIAKCTAAKRALRYLRDLDKEREAAKKASAHH

>DRH-1 - L596_g26385.t1

MNTHLKTSHPLSVINWDKFTPETPVAIRPEHFDLRPYQEELAHHALKGENTIIWAPTGSG

KTIVAVHVIAQHLLKGNGRKVCFVVPNVTLLEQQKRVCERHMDAKVNIIKAESKAPFSGM

VAASHIVLLTPQMLVNALQNTTEGKENFSLSVFSMIVLDECHHTAESHPYNVLMHHYHDV

KLKGTGKSPQIVGLTASLGAGTSRNANEAFRHIQKLCANLDSPKISTVEKHKADLTGFAV

ETKDRIRYVESNYRSKPFFKDVMELMSELENGIFVHPAIACDPVKKAMIKEQVCRSSDAS

STANYRSSASTRYSQAYQNWLGNALMITLPKVDLPSDARVEIMTRFRLLKILYKMIELWA

NFNGKCAMDYYMKESEGLPIPLPVHDRIRELVSYRNDNSDMLIEMCQLLVEKFGEGTDGA

SPARVLIFVKEREYTYMLANIIDCCNELKALGVRPDFLISTNSGDGECRLNPEEQRSKLE

KFRSGEINLLCSTSVAEEGLDVAECNLVIKYNYASSDIAHVQRRGRRARHQNSYAVLFTH

DKNLEQQENKNILSEELSNQAIALLRQMPQKMFEAEVQELVRKSYSDRVIERAAKETASL

ALKDVGARTCTEFIGRSTDVRHSNHSLHILCDGSIWDRFTCEESVSQENFLKTSELPIGK

IYCNNCKEVWGRIVIFKGIAVPIIACKGILLQNARGERFPCAKWAGIKGRYFCPEEVKSV

DLARLSAAPHRPQFLTVLMGETESRSL

>DRH-3 - L596_g25860.t1

MTSNGTRFELDYVLTVITTLDFILEPFFLFLALTKSTPSMWFYRIFLIAISLCNCAFSIV

FFLLSAKFIPIDDAVCLISSHFALRSGRVLWQLSMFFMMIQFQIVLLMLIYSSYEISHPL

RPLNWRSKAVILAVFVFIFLPASLVLILEQQVIMGEIEEEKREKVPLSHVRLFRKEFLES

GRINEAFIDKCEKFLSFDVVEILKKLFTINPGLARKFFFRQIRLKHPKESGIIIPIIERC

YGDDTEVLSELRLENLKNASKERYLKIFSDDEFCAGDTLSTQIRPQPFIKNLAAKYGNRY

EKMIKKVEALLKDKEHDAAACQILRSMPKIADDDEDDSWWLHLLDICDMDPHNRACLVLL

DVDYRNIIDMFRAEQRAKRTPAIQGKDEVMFDETISDLDREFRGQEPYMEHRETVDENLI

DDPDKIVLRDYQEEQMEGVRKGNTIVCAPTGSGKTIIAAQTLLEHFKENEKARAVMFVPT

IPLVEQQAQLLTRFLRKRKYILTASGAERVVSLGRKMLSADVVVITPQLFYNYMTDPRES

ERIYISDFTLFIFDECHHCDSNHPYRKITKRISAFEGKKPHVVGLTASVGVGTKTMNPQE

AVDHMIRICANMTAQSITTVQRCLKSLDEKVRIPDDYIRTVHRDLESPFRGKIAEVLGKF

VYIAEEKLKNTVLAGGEFESFPDKRKVCQFFGFVKRLKNELERNQKVEDRNALIYTLARI

EVLYKLFNLSDLLPARYAAHVLKEFNDENKDGLQEKYKHYLRELGEAAIKNEERDMNKDI

LVTLKEILVDQYQKDPFSKTLIFVSTRDLARDLSLYLRDQWDKYGLPRKVKPTREGEDSE

KTYKPVSYITSSNQATSAGGLTAQQQRETVAHFRSGHHLVLVATSVADEGLDIAECNLII

KYNTSESSELKLIQRRGRARAKQSSSILLALDGAIEKAEYDGMLREKLMYAAIKHLHTLP

DTHVYRLIEAKAQQLLVDEKEAEERAKQRKEEASEEVWIVACKKCSEQLTTTGSLRNAGD

KRVASCDPSIWEKLKIVGNQKAESHGNASLEISVAVCSNGDCDNTIATLGTDGNTFMPFL

RAQSVSLYRQAEYDADNREGKQSLKKWRSLEAGEYNIVLRSIDDEDRKRMCSSWESQNSE

KAKEIVQKRQDQRAFKRARYLTKESRNRELAKKVILKQKEDPNAEEDDYGYSSIADDKIR

GEDLEKVKIKAEDFAQVLHSEPNYLSAMKGYEAEVSDNSDNDYE

>DRSH-1 #1 - L596_g13580.t1

MEDPGCSGTQKLFEGAKRTLDKREDAQENEKTENKEQLLTESDVFKRLAEIDLTTEESTA

NSPDPSKIQDLSKFTVAVSKRTISEPDASEFYVKTENGDTIGTDRLKTVHEAMQFRVLDV

IDMARREQPQSEPPTMPTFHENCKCGHVSESEADSSSGSESEEDDENADLSNVSDANKHS

LVAKREIARKQQHPAGLSQEMCFNEPGQMNDGPACRCSWASKQSGIRHSIFVGEERVEPC

NPMSSNLRNLHHYFMKVTPNPVEQSRNASLIYHNDKGYVFEGFSVFFHRKLPQFFPQTPV

NKLSQEFEVVFIEEKAPEQFTVHDLESFHTYMFDHLLEMYDLNRRAKDVFDGCPVYHCMP

RFVFKDYATEAVEVLPMSAVLHHIAGAFQPVFDDRLVQKIRRDENLFHNMSSHMKGQIFV

NPSKRPATIRVDLIERQDPYTKSVNKDFHPVLTHIGLRPSVYTFSAKPAYQQAMKKYLRT

RHLMSLQGKLSYEDKQKLVEQEANIRKLKAEAQHKRDMVMSVTSRGFYSTTFYPDIVQHA

VLLTLACSHVRYHWCLETFEKRIGYSFKNRTLLELALTHPSFRANYGTNSDHTRNALANC

GLRIDKARNDNRNSQVDRPSRKRGYENLREVMSMKGTEKAVLSPVHHNERLEFLGDSVIE

FITTIHLFYMLTDLDEGALATYRSALVQNKHLAVLAKKIGLDEFMLYSHGPDLCHESDFR

HAMANTYEAMMAAVYLDCDLNECDRIFADTLFMDEKEEKSKEKLAWTKLLDHPLKRDNPY

GDRHLIPKIDSLQLLTQFEDSIGIKFKHIRVLAKAFTRRCIGYNNLTHGHNQRLEFLGDT

VLQLVTTEYLYKHFPNHHEGHLSLLRTCLVCNRTQGVICDDLAMAKYLVIPPNSRKHTHV

MSIRWKERADLVESFIGALYVDRGLEYCKTFCKVCFFPRLKYFIESQRWNDPKSQLQQNC

IALRDGKNEPEIPEYRVIAIEGPTNTRLYRVGVYFRAVRLADGVGHTVHLAQMNAAENAL

KQHADMWPSMSTKKTEKKASHNNWERASYRREGNSAKYERSSNRQGGGNDYRNDLSGQDY

RKVPDGRESRRNSQNSDAPRSTFNAPYMQQNRRPYDNGQEKPHRSRNGQQYDDRSNQNDL

RNGRNHHAPSYQNEPSRFGPTRHRHDHEFRKPYDRPQHDFRHFTNDRRPHNESQNTSYGR

DQNKGEAYSGVRSRYYESQSERPPYRPSGSQKDSNPVYQRNAQPYRQQNNEQNSCRQPTW

QDSQNKPH

> DRSH-1 #2 - L596_g13580.t2

MPRFVFKDYATEAVEVLPMSAVLHHIAGAFQPVFDDRLVQKIRRDENLFHNMSSHMKGQI

FVNPSKRPATIRVDLIERQDPYTKSVNKDFHPVLTHIGLRPSVYTFSAKPAYQQAMKKYL

RTRHLMSLQGKLSYEDKQKLVEQEANIRKLKAEAQHKRDMVMSVTSRGFYSTTFYPDIVQ

HAVLLTLACSHVRYHWCLETFEKRIGYSFKNRTLLELALTHPSFRANYGTNSDHTRNALA

NCGLRIDKARNDNRNSQVDRPSRKRGYENLREVMSMKGTEKAVLSPVHHNERLEFLGDSV

IEFITTIHLFYMLTDLDEGALATYRSALVQNKHLAVLAKKIGLDEFMLYSHGPDLCHESD

FRHAMANTYEAMMAAVYLDCDLNECDRIFADTLFMDEKEEKSKEKLAWTKLLDHPLKRDN

PYGDRHLIPKIDSLQLLTQFEDSIGIKFKHIRVLAKAFTRRCIGYNNLTHGHNQRLEFLG

DTVLQLVTTEYLYKHFPNHHEGHLSLLRTCLVCNRTQGVICDDLAMAKYLVIPPNSRKHT

HVMSIRWKERADLVESFIGALYVDRGLEYCKTFCKVCFFPRLKYFIESQRWNDPKSQLQQ

NCIALRDGKNEPEIPEYRVIAIEGPTNTRLYRVGVYFRAVRLADGVGHTVHLAQMNAAEN

ALKQHADMWPSMSTKKTEKKASHNNWERASYRREGNSAKYERSSNRQGGGNDYRNDLSGQ

DYRKVPDGRESRRNSQNSDAPRSTFNAPYMQQNRRPYDNGQEKPHRSRNGQQYDDRSNQN

DLRNGRNHHAPSYQNEPSRFGPTRHRHDHEFRKPYDRPQHDFRHFTNDRRPHNESQNTSY

GRDQNKGEAYSGVRSRYYESQSERPPYRPSGSQKDSNPVYQRNAQPYRQQNNEQNSCRQP

TWQDSQNKPH

> DRSH-1 #3 - L596_g28273.t1

MTTSPDIELFNISSDEEDDLLDFPKMAPARKATQAPSREKHCQAELSRACEAFSVQTLDA

IGVARCRAADGNAAENGFETLLGCPCPKSQLGTGLRHGFYPKEECIPACDKEQINPPNLH

HYVLKVDSPNVPQVTRIMHGNKEFVFDGFSLFFHQKLPVELLCTPLKIALGTSATVTLLP

ARSPKGFNVKSLDWFHKYLFEEILSFPMSQPNQNQCSFYHCLPHFVHKEGKNVHLLPMIA

VLQHLQANFKPIFGVNDLFKNHRGENVVAKIRGQVVFNPTKTFSALRLDHVEDPRDLLGQ

PKLVSFTKWKKSVHTRSRTKRKFMPNRIKSDQTERPFFENAVPSKGYYKTGLYPDVIQHA

LLLVPTVNYMRFHFNLQILEVRIGYTFKDKTLLELALTHGNFRGSYGPHRLIVKRQNETF

GFRTNLDTATKELKRPSSNGSKREPGQTYERLEFLGDAVLGFIAATHLFYMFSMADEGTL

SLFKSKNVDNSRLCDLAKRSGLQQFILYECDRNLIIDEGSHPLLANIFEALFGAIYLDGG

LNECDRVFADITFRNEQNGEENKLAWKNMLEHPLKRENPHGDRHLIPKCKSLLLLTKFEK

SINAKFKHIRVLAKAFSRACIGLNNLTHGSNQRLEFFGDTILQFLTTEFLFHKFPSHSEG

ALSLLRQGLVNNKALSTICDKLSMRTYIVVPEDMEKMEHVWILTEKSKADLVECMKRWKR

KAGRTRNPPSKKRSTSSAEALDAQIYLDTKFSATRDHQIIDLTMSPSTSGTFNTLKLFKS

LFRGKQLGVGKERTIKKGEMEAAKNALESITEAVFKDMKRKLLEEKKIDVPRTGVCSYPS

L

> DRSH-1 #4 -L596_g13580.t3

MEDPGCSGTQKLFEGAKRTLDKREDAQENEKTENKEQLLTESDVFKRLAEIDLTTEESTA

NSPDPSKIQDLSKFTVAVSKRTISEPDASEFYVKTENGDTIGTDRLKTVHEAMQFRVLDV

IDMARREQPQSEPPTMPTFHENCKCGHVSESEADSSSGSESEEDDENADLSNVSDANKHS

LVAKREIARKQQHPAGLSQEMCFNEPGQMNDGPACRCSWASKQSGIRHSIFVGEERVEPC

NPMSSNLRNLHHYFMKVTPNPVEQSRNASLIYHNDKGYVFEGFSVFFHRKLPQFFPQTPV

NKLSQEFEVVFIEEKAPEQFTVHDLESFHTYMFDHLLEMYDLNRRAKDVFDGCPVYHCMP

RFVFKDYATEAVEVXXXXPKLQLLLLVTVCAFH

>EGO-1 #1 - L596_g919.t1

MLAAENDRPPTSDDSQMELKEGGTAVMDVKARDAPSKKAPRVFPESDDSDLVRIITEAER

DRRMQLKLDKADFQKKVIHEMSADIAGCSASCKRVSEVPGNVNGVPKSAHFEVELTAPEW

DRAFFPMVHNFLQISKDYAETKKANALMQVSNPTLLREDFEPTNKLLPLQSFAFGCMRSP

FEFTNHHEFHSSESNAELLHDYFNHCREEPNHKEAMTIDFNHDLGDSFVKFASEEGNGYF

VYMLKLIYNKVRRIIVNFERLDSTTSTVHGVKLFLLFNNPIEVRRSQKIKGRKGDSYWRF

DTNGDRCLTWNNNEERQAIIADCLNLKLQFEKLEKNVLYNILSRLRRRCNVILEFSSVQE

VPFKGHVDRPMFGDHAIARKLKEMDKYDVNYLIEALCSRGAVINDQFLESEKTRDDYVEM

ILEDYERNSFITIAALERILNIIDEHLEVRDIHLLYRTCFNEEAAHEKDLKETEERRKAD

GFLKVRKIVITPTRVLFVVPEMMMGNKFLREYDPDGLYSLRVAFRDDSGLKMNNSNVGSQ

LVEKTIRESFKGVMVAGKEFVWIGSSNSQMRDHGCYFLLNQTRDMRKILMEKTGEFDEKD

PIKAQARFGQRFTQARAVPFDLSRDDYDFFDDIKGGEDENGERYTFSDGVGSISVHFARR

IAEALKLQNFVPSVYQIRFRGIKGIVTLDPNLDYYRDHAKKYGIQERRKHHRFDLQIAFR

KSQDKFNARVDVEIDIVKFSTPTPMQLNRPMINIMDQVTAMQSEASHRRMCARVHQLLDQ

QLSDLGKCLVDEHKAREKLFDIPRRVDVHLLTLEKGFHLTEEPFFRSLIQCHVHTTLNRL

RAKNQVQIPFNKGRMMFGVIDESGGLQYGQIFCQVTNNLQLKTPGVTAAKTILTGPVLMT

KNPQNVAGDMRMFNAVDIPELRHLVDVVVFPRYGPRPHPDQMAGSDLDGDEYAVIWDPEL

FFDRNEEPIEFPKPKKPVPPADADSTDLVINHIVNYIQQDSIGVISYAFLVNSDLYGIDS

EVCKRIARKHSMAVDFPKTGVPPQKLTREPDEDNNVPPEKPDRYPDFMEKSGRDPDYVSA

GLNGQIYRRSKEIQGILQFTVGHQESRAPEPDPDFLVEGWKTYELDAREAMDAYNASIRA

LLDNYGVKDEAELFTGNISNARNRLSDKDNDDMSLFNTNYAIEQRVSTIFQTHREQFFDK

CGGYMELTVSDMTFIPTAPKINSRDLERRLCTEISPEMKKRASAYYQVCYTLQPKRESRR

ILSFPWIAYDVLAEIKKENYARKIRNQELRIDIGRPIMSRLDDYIKEYLKHPRFAAFAKA

LVFDKVCHRYATVYPALTELFFVLWQFGFEQKIFDGCILPENLFYLFLLFAVSTENPFLE

KIPADANVKAMEKRDLHARIGGMGGPLMDFLEYLCTREFQSQLFVSFNEIGVDDCLQGAT

LASLSRAAIHTYYRITFSGMFFALPQLGADHSKNPSTKEQKFDTSGVRVYEIDPFVIEVP

SFAKEEGKLDELTQMLKLRTGMQHLVVRIKFIVRKTGNLRLLVSGIGSLEAIEALRDVVS

IKPNLRSGSATVVAKAHLMADMVYAKIMGSDNFGGGPFGYCHDISDIKKATPRKKPIRKD

LRN

>EGO-1 #2 - L596_g10626.t1

MSADSDSSANAKTSGVIRIFLDRTYERYDRCTELQHYVLEKLTREETFQTFTLKRNGLPR

HHKGDYEERLSEYELTRMPPPKNADKSKEPWDREEYPPRKRLLQLVTEFTEALSATMKGP

LPAIMQISDDFFLKKDYDPIDENLRVAEFALGNLVDPFTFHYSELKSSTDDKAQHDTHIL

RSFMKDNNIKSGMIATFEHDKNILWIRCVHWQKNPQAVYGVSLKVPYNQIRRLIIDVDKK

ADCATIYFCLNYPVEIRRAKMKSQKGRNMSAFGTEFDRKGRDGERCLSWEGEDSENGRLK

QAIADSPVLALTLTDYKDEGFYNVLSRLRSRCKVPEFTHVHKEKRSDYVDPPDVLLHLRT

KSGKLNDFSVAYFVQAVFSRGALVKDALLRSEDVRNKFIAKICKQCDKNRIVAVCALERF

LNAIDEKRELRDPIKLWDFLVNQVSGDIGTIQKQLDKNEKDGYTLVRKAVVTPSRILLLT

PELIMGNRGLRDFVNNSDDVIRVLFRDEDGTQMRKISVGEIIIQKVVGQVLEDGIRIAGR

HLCYLGSSNSQLRDNGCYFFEKSQIPDIRKKMGTFKEMSVPKRMSRMGQFFTQAQRVQRP

LERKEYRESYDLIGGADLVNEPYTFSDGVGVMSVALGEEFSRSLKLNGCVPSCFQIRYRG

FKGVLSVNPMLDEIRAWAEANLSDEDIPKYRNNVVFRSSQKKFDASKNAKLDSAPFEVVK

YSAPCQISLNRPMIDILDQVSEIQSYKAHERIWSRIDELFEKQVAKAASSLTSEHYARER

LAELPKRIGTSDLKEEDGFMLTQEPFFRSLIMSSVRLSMSKLRKKNNVDIPSNLGRLVYG

IVDETGQLQYNQIFCQVSSSIFVKHPKKSALKRILKGPVMMTKNPAIVKGDIRRFEAVDI

PELRHLVDVVVFPRYGPRSMPDEMAGSDLDGDEYVIIWDPKFLFDKNEAAMNYPRPLKTF

EKVREDEIEKKSCEFFIKYIIQDAVGMLAHAFLANSDYYGIESEVCENVARKHAKALDFP

KTGDQPDALTKMPTKSEIYEGKQIPQEKPDRYPDFMEKDHQRCYASIGLNGHIYRRARAL

DDILHRSVDRHLSDKIEVDPDLIVDGWEDYEDEAKKTMNDYNSQIRSVLETYGIEDEGQL

FSGAISKMRSGVGDATQDKDLFGAFNVLSTVSKHVALIFDEFRRSFFDEHGFGGYLHCTD

LKYETVGRSDEMHHSYKRTCRKPTLEMKKLASAYYKLTYEAARQESQQAVKLLSFPWTVW

DVLAAIKHDVAEEKRRSSDRRDQYMNRRVKPFANLLSEHIAKFCRQRQEDLDDFKNLLVN

TDGIIALMLERYEGLVSILFFVCVWADDSNVFSDFFNYQHLCWILILFGQGIYPWNHAKT

FHTDLPIFYPIDKLKEVQNIGKESVNLNHLLGGIGSVILLFLEFLSSYYFGHKMKSISFN

PLGLNSSLGSCYLQKLFHAATKTYNEVIFSVSFDTLPQPEEGKVHRSLREFEIEPFMIEL

PLQKESEGSEARYNLSDVEDQIKEMTGASHVRVRPLNDVFLSGVTRLMVSAKGTLESSQK

LRTLLSVMPSCRNEHNAKWGKLFMSERLLMRLNLVHS

>EGO-1 #3 - L596_g1037.t1

MHEILFLLAEGFVEGPVGALEGKDLTVFFSKSFALNFSWIKSPPVTINRLTLCVHQIDSM

LNDETKALARLSELTRRIDFNHLTSEKGFQFIDEPFFKSLIQCCVKFTLSKPMGRFKW

>ERI-1 #1 - L596_g16241.t2

MRDRLKRRLRKDLLSTREYEDEFSRNKLRQHFDFLVIIDFECTCEDEVVDYPFEIIEFPA

VLVDTHQKKIVSRFRTFVRPKVNPVLSAFCVELTGVTQADVDKAPRFSEAVLKFHRWLFS

HVYPSGQHDFGGLMKSYAIVTDGPWDLGKFFQLECIRLECIRSERYNAKIPHHFRAYINI

RRVFTLKYKKTHALSKVNLAGMLAVLGMEFEGREHCGLDDAMNLARIAIRMMEDGAEFRI

NEKLVAAEYADKYTSFVPTISTTGMSTAERDRRKWRLNLPYRIVNICKDRFMTGAYADCD

SCDEDANFYEKAANRK

>ERI-1 #2 - L596_g986.t1

PISAHNFRGTSQQMRDRLKRRLRKDLLSTREYEDEFSRNKLRQHFDFLVIIDFECTCEDE

VVDYPFEIIEFPAVLVDTHQKKIVSRFRTFVRPKVNPVLSAFCVELTGVTQADVDKAPRF

SEAVLKFHRWLFSHVYPSGQHDFGGLMKSYAIVTDGPWD

>ERI-1 #3 - L596_g8761.t1

MSLEELSALISEASKRKPKRAKRGEEEAPNGRYEDPKEFPAVLVDTHQKKIVSRFRTFVR

PKVNPVLSAFYVELTGVTQADVDKAPRFSEAVLKFHRWLFSHVYPSGQHDFGGLMKSYAI

DDDGLWELRNFLQXXXXSILTGLSVKKRNSAFIVQVRTTYPMHTASTDGLAKMSWGSDEG

SISMAIAALRKSRPAAFELEYPDLMAKTKPTPLPRQPSPAQQVEPKSPAQSIAPTSSSED

LHAHLAKIQVTQPSVYLPSRPAYPSVAPSVPTPEAVVPQPEPPRGPPGFDRSAKPKETTF

DTRVMGGASQTDVQVVPSREVPHVPSLPVAPARPRVDRSTKEAVARKEAEFQRNLFAVYE

ACTQDFRNMT

>ERI-1 #4 - L596_g16232.t1

MLAVLGMEFEGREHCGLDDAMNLARIAIRMMEDGAEFRINEKLVAAEYADKYASFVPTIS

TTGMSTAERDRRKWRLNLPYXXXXVYCGNAIINGRLIRITFKFSRIAWSQHLACEGVTKI

EMLAAYVAFEARRRLAQFVTYRES

>ERI-1 #5 - L596_g16263.t1

MSVSVTRIQELPSVLRPFVPRQSLPSFSRDGRTPETELRSGKTRGRLLRVRGRRRGRRRG

FRRRRVRLQPGLRRRSDDLRGTLGAYLRSLETDAEESEKRGRGCSKWPLMIEDPNEPLRC

EGYQVPKHDFEFPREVSVXXXXKANLQKELEELQLNTGGTSQQMRDRLKRRLRKDLLPKY

KDEFSRNKLRQHFDFLVVIDFECTCEDEVVSRFRTFVRPRVNPVLSAFCVELTGVNKAPR

FSEAVRDEFSPGRESDEGQGRVSDQREARGGRVRGQIHRVCPRNFDHRHERRGAGSQMMW

RLNLPYRIVNICKHRFMSEAYADCDLYDEDANFYEKAANRK

>FKH-5 - L596_g10448.t1

MTPRGLCVCNLSSQPQHSMEPSSESSRKFMDMISTLCAEVDNKQKLPEKKPQRNASGKCM

KPAYAFSAIIALALKNSPDGRLPVWKIYAFILEHFRYFRTANEAWRNSVRHSLSKNGNFE

KCLKEDGSKMKDGNMWQIIESRREAIDREILKYRLSDKYHAMNLAALNKPEILTSLEEGS

FGLPPFVFEKEYKQAYRILANQKNKRPEIPKNFEVASNVFKESNMNTLNTDLDTLARPNP

ISVLLNENTPSPISRIDLLKPSIPSSSSQNMSEASMNAEEKQIYEKYLTSDSNPLLSSAA

LPGAVVPEIDDCMLWECEQDTLEAGIQTRLRHDENDFWLPTRSNCIFDLPSDNLSVVSDG

F

>PASH-1 #1 - L596_g24740.t1

MRKHKKLKPRILLAKTWCSPSPFGDLXXXXSCRKEKSLTTPTDHLIEDSDSDGGIRNRSF

GETQDQPQTTQTDHLDGSRSGDEFEDETLEKTPNKSHTSKNEKEEQGTSKRVLAYSEYGL

SNKLPEGWVEVSHESGVQVYLHRMTRVCTFSRPYLLGEGSVRHHKVPQTAIPCFFQRKVR

EQLEQREKEIEEVVKSQKGAETSAHLIAKLQAPDVKTIAQSQLTPDDLFDYAKSVFEFKT

ITVYRDKASKWRHLKEQKRAEGKINAAAKAALLDSAPEIPQNMKIITIALDVESEKKAVT

IDPRAKTSINILSEYFQKVFRSTPRFTDELTSVPLKEKIMSFQNLAKSELNPEIGDEVIL

GEGVGAKKKTAKLRAAAEAASFLIPEIKFDLDGKVISWNEDSGNKTEKDITVDFNQYEIT

DPRIAELCKSYAQPSPYTVLQECLRRISACSNDEIQIKTKHKNYSSFIEFTMAVGEQKVS

VTCSNQQEGTQRASQLMLALLYPDLQNWGDVLRLYGEEPQRVVKDRSKEVTTLQTFVQNG

GDKVLSDHNNAILDKLKEEMKATADGLDLQKGLLKEVSASHLPISECDAVLSAVYAEFHR

VKREVPEMGI

>PASH-1 #2 - L596_g24745.t1

MGDVSDDLKLLMLERERILRELNSQNPGYDPDDVEPPPPPPPELPDEESSLSSAEDMYPP

PPPPFTESFPSPSCSPAAPVCLTASPANPEPCTSFDSVSRDPSSLQRDSSSTALPATPLQ

APIKKDAEESFDSPVAKRPRMDGCPFSGTQFSEESPAVNDRKSTYNDDNVVSDADVSNEA

NNSQAQSVENSGDSDLSDDSDDDFVDGLLEKTLDESRTVKNEEDLEIKSKRVLEHRGCDH

FDVLPEGWVEVSHESGLSVYLHRKLRVCTFSRPYFLGTGSVRHHKVPQTAIPCFFQRKVR

EQVEQREKEIDELIKSHKETESSAHLIAKLQAPEVKTVQDFEQSQLTPDDLHDYAKTVFK

FKTINVHRDRTWASHRMRIKDKKRADAEMNAEANAASLDCDRPVLPSDVKLITIPSLDQN

GKPQRKNFLMNPQGKTSVSVLHEYVQKVVKSTIRYHYEETRSSSNPYIAIAYLKLGQISG

KATSAVSIKEKIMLLHEQQRREGLKNSSENTDEVFLGKGSGVSKKLAKLRAAVEAVSILI

PGIEFDSEGMVVNSKKETGTENNANEDVISMFNKFDITHERIPELCSRSGQPAPFLVLQE

CLKRYSASADTKLSISSTRQKHHRHEFVMSVGKHETKVICTNKQEGKQRASQQMLQLLHP

ELKTWGEIIRMYGYEAQRQFKDARKKGNDVTKLQALAADGSSKVSLNPNAAILSRLNEEM

RKTAERVGQLKGPFRPDLSNDETAVTDWEVALSAQYSEIHRDRVEVPIIDL

>RRF-1 - L596_g7841.t1

MDDMSMFNTNFVIEERFSKIFQKYRLEFFEEFGGFEACTVDESSLVNRSKNLHANKAESD

MDRRICKNPTEAMKDKACAWYNVCYFYANKAKRRYLSFAWIVWDVLAEVKRENHFKNNRE

QRLMGIPIHTRLHAYIEKYTGDVSNRVALEELKKLIAKEERHIAKYVEAHEGEIWIVNCS

>RRF-3 - L596_g11915.t1

MSSKAVSTRGVAFALRLSVSQEGLPKEILLHAARQMLQSCGCSSVRLETPVQALQAEYED

CRLEISGTVTFAVPSSVDAWQMIIVFTSKFCAETGMGLELQPSLEMDAFDRPNHDNCHIL

WFAFGNMPNEGLFLTRGDYISGYNKKSNRFVPDRGYNVNYVAGTQLLLSWANFEHDRKLL

TIYFAVQLPCPASDGLLFKGYKLVFTYHNIISVIADTDDSRAGNNVVYLKLRHPPQLWEA

IPRLYANRRLVNLEACRDWIRVFEFPGSNRFYGCTKSTLGSSSVFAFGMPKNVVDPKILF

EEEREEWKSFAEDLTTRENPTRSLYDILSRLKRKANIRLYFGSILSVVRSVMRTCDLPST

DSFRVNYCLEALASRGFSVMDQWFPIDNQEANYFPVFFSRVVWCLGECKEAVENTLENML

SIFDERKHHVNMVTVFEYLYEQNIKSLVEERDMDDCSYNDLPTNCVMVRKIMVMPSRTLL

MPPEVMMTNRVIRQFGEENALRCVFRDDGGNKLVPKEFTRGRSVEGQSVTIKEIVKGTLS

SGIVISDRHYRFLAWSNSQMRDHGCYMYADIITQDETTGEEIVQDITTIRKWMGDFSSSR

NVPKLMSRMGQCFTQAQPTIRLGPSHWCVEKDFFTGPFGNPTKYCFSDGVGRISLRYAER

ISNLMGLTFSPSCFQVRYRGFKGVLCVDPQLDRTSDTPIVFRESQMKFIDYERGSQGPVL

EVVKYSMPSPVCLNRPLIMILDQVAEKNGSHCHRRVCSAIHQTLENELNELAVMLYDESA

AIRALSQRVNLSIDFGQFVNTGFRLTEEPFLRSLLLAIHKYNIRQQLSKVKIPLPNGMGR

TMYGVLDEYGVLQYGQVFIQYSQSINRAGQRKILHEGPVMVTKNPCHVAGDVRMFEAVYQ

PCLEHLVDVIVFPRYGPRPHPDEMAGSDLDGDEYTVIFDTDLFFGSNEPAMEFPKSEAPE

FDVMPETDDMVDFFLKYLEQDSIGRMSNAHLMMSDKLGLFHEICNNIARKCSVAVDFPKS

GQPAEPLQMDEQCADCPDYMKSNTKPSYRSKRLIGQLYRKAKNIEDIIDLMPNPGTDRNI

PFDEDLNAEAYLEKNPDVLRECIRVRNAYNCKMQQLLDEYAISDEASLITGHIISMKRLT

EMEKDDYTFYHTDRIVELRYGKIFAVFRKMFFDEFGGEEGHFEIHTSGLREFKMNDILIR

KACCWYTATYGKYGVHKGGIRFLSFPWILWDVLTSIKKQKTLQASRICSVKSPLADELSK

IALFKCQNEEKAFVEFCNDIQKAVPIVKTYRENYGDHFLKACYVLNYWLLKERFYERTGM

TSQQLVIIFLQFGIAIRHGTLNNYTGRIPRIFPKMITQSKDIIDIDSEGPLLPEIGLQII

EFLRYVSGYDFMFSECIDLSLGNILESTRLITRNTIWKSFSLASFKVFHHVTLSYSFRAL

HCGSTDVGNPEARQVAWDYGEIDNPLIVHKEALLVNTPGMPPVHLESTLNILQAWSGVDD

LMVRPMNHRDLFIVTCAGGAESRQMLRRLLQLPPTVLREALLTDSIPSEIVTGTEM

>RSD-3 - L596_g5786.t2

MSCVIDSGMDFTFIPSTFAGRRSVGAGAMSDLFSGIANLTKSVTDTFNTYEIRKLGDKVQ

GYVMNYTEAENKVRDATNEDPWGPTGPQMQEIAHMTFQYDAFPEIMNMLWKRMLQENRAA

WRRVYKSLILLNYLLKNGSERVVGNARDHVYEMRSLESYKNHDERGKDQGVNIRHRVKLI

IELIQDEDLLREERKKAKSEGKEKYQGYSKEEMRMGKGGSYSSSSMGNIDDWNGRSYKSD

NFSGGYRDEPSREVNSFNFPDDERNRSESPELGIRETKPVEDEDEFGEFTEARSTSNSIP

SKSDGVIPPAIQGPGVAVHSPIKPPQGGVHLSLDNDPFGDFTSAPIPKTQPEVDLFGDFS

TPAIDAIPNLPRPPTPGYSAPSNSNQPSSTLVDLFGDAVPSNPTPNAAAPQVDFFADFSN

PAPQAPTSSVAANDFFASIPQMTNVTPNQSFVANFDSSVQTQAVSSNLDLFADVSLLSSP

SHPSTNAVPPMASPMLFQSQSMTPSPAASSSHTPRSVASANPSKASSSMWDDMKGRVNID

LDNLSLRNSGNMKQSLSMNQMQKNKNQGPLF

>TSN-1 - L596_g28061.t1

MTDSAPQQTPAPAGAVKRGTVKQVLSGDALILQGPAVNGPPKEITVYLSNINVPRLAKRP

AEGQPVSSDEPFAWEAREFLRQKVVGKTVSFVRDFTATSGREHGRIYLGGTSIENAENVN

ETGVAEGFFEVRTGKQIDEYAQKLLDLQEQAKSAKKGRWAFDEQQLKEKVRNVKWNIDDL

RNLVDTYKHKPVKAVIEQIRDGSTVRAFLLPEFHYVTVMLSGVKAPAVRLGSEGRAEEYS

EEAKFFVESRLLQREVEIILEGVSNNNFVGSVIHPKGNIAVLLLENGLAKCVDWSIGLAT

GGAPALRAAEKLAKDKKLRLWKHFKSVSSGDKKSFVAKVTEIGMGDSFFVQKDNGDEIKI

FLASIRPPRNEAGQEKQSVGRQFRPLYDIPYMFEAREFLRKRLIGKKVNVCVDYVQPKSE

QFPEKTCCTVTVGGQNVAEGLVSHGLAKVVRHRGDDENRSSHYDALLAAEAKAETGKKGM

FAEGDPNEKGGVIRVQELTGDANRSKQFMPYLQRSTRPEGVVEFVSSGSRCRVYVPKETC

IITFLLGGITCPRTARPGPGGKLIGESEPYAEEAMKFTRSKCLQHEVQIEVETMDKAGGF

VGYMFVPNDKGGHNNLSELLVENGLASVHFTAERSHYYNQLNAAEERARRARLGIWKDFK

EEKQIDALEQENAENVERKLNYKQVAVTEVAKDLLRFACQSYEEGPKIVQLMRDLQQEMN

ANAIAGSYTPRRNELAAAKFSQDKQWHRVRVEGAKAGMVDVYYIDFGNRETLPVDQMAAL

PSKFVTQAPGAQEYQLALVGVPNDPHYAAETIAAFENLVFSNSNILLNVEYKAGNTEYAT

LSIDADGTKSDIGKTLVMEGNALAEQRREKRLQTLVSEYTEAEQKARRARKNIWEYGDFT

GSEV

>UNC-130 - L596_g11813.t1

MRFSMDSILCPPSATPDNKLVAASRKRLLTQSLTPSPPPVESKMAKLDSEDSSSSTSSED

RQPPAVTQIVVAAIDKGELAAAAVLRSDGDSSGGEELATEAKQNALAAAAVVGQHARGGG

EDTTTSPGTSRSPVTSDEGDSGDECNESSNGDKRIGMSSTRSRSGATKPAYSYIALIAMA

IYNSPEKKLTLSQICDYICNRFQYYRDKFPAWQNSIRHNLSLNDCFTKIPREPGNPGKGN

YWSLDPNAEDMFDNGSFLRRRKRFKRQSSNNDFASLPFAPPGAHFLPPQAAFLANPAFVL

RSPMMPRVGPHLGLPPQLYRTPYSPGFLLPPVTSTNGLPHGLPLSLSSLPPGMDHQRLLA

AMAAQSAALSSASSPPNTVSPPSPAGAQAH

>XRN-1 - L596_g18210.t1

MGVPKFFRFISERYAALMERVQENQIPEFDNLYLDMNGIIHNCSHPNDDDVSFRISEEEI

FGNIFQYLDQLFSIIGPKKVFFMAVDGVAPRAKMNQQRARRFMSARNAEHQQKQALAAGK

PLPTSDRFDSNCITPGTSFMIELQKQLEFFIQMKQSTDAKWRDVRVYLSGHNVPGEGEHK

IMDFIRTERSKPDYDPNTRHCCYGLDADLIILGLCSHEPHFALLREEVTFNRAGQKKDKV

GIEGTKFFLLHLSLMREYLAMEFQDLKETTTYRKSNTDSLPFALNEENVIDDWVLMTFLI

GNDFLPHLPNVHIHEDALPRLYKAYKAVLPSLGGYINERGILNLKRLETFFERFSVIDRD

NYLDQFEDADWMRNKKQREQGEGPPDVIPQVVFEGLLEEEDVIESSSTCVSSSDIGAFDT

DSEDERKEVEKITGVKKKKNGGDVIPSASEALAAGGWDSESSLEIDGLNLEDSDESPDEN

WNIVIHRSFKKKRRDYYAEKLNYVNISAEELDDQARGYVRALQWNLHYYYHGCMSWSWYY

PHHYAPYLTDVRNFGDMKIEFDLGEPFNPYEQLLAVLPAASCRCVPPALRPLMTESSSPI

SAFYPTDFKTDLNGKRNDWEAVVLVPFIDENLLLRTAKAAYPSLTEAERRANTHTGDLLY

TYSTKDLGELNSSCAKFDRVAENHSKCENMEKNAFRLPRSKIVTGLLPQTKLDVYFPGFP

TTKHLNYTGSLEIAPVKVFHMPTRKPVMILKIGDNTENKYSASGVDPRSLLGQEVQVNWP

LLKLAKVCSVVTPDGRYLMENHQTSFTDFGPNKHKVFKDIKDMVTDREFGRYGICVSNVK

AIVEVNLFTGSRMKFKGNNVIVEKSWSDDVFAVSDGLVLQNVEVKNDMEQKFKSAADAFP

VGSKVFLNSFKVSAYGMMGTVARNDVAARGTCLVEGVAPLQVNVKAAVSKKQYKKFWFSV

FDLAKIIGQDKHVVNRVTGTVIVQMDSDFKDEEQEERGKHHHKHGHERPRNNINVGINLK

YTKRNEAIVDFSKRENETWYYSLFTLKVVQDYAQRFPEVFRALRQNKDQYLLEDFWPELS

PEERTNKAKEVVEFLKALPCSSRRPETCNYKYADPEELLLIQKEIAATVAETNVQQFTVH

ARALFRPDFVTGDSPPVPNVEFKILDRVIVAKTHRYVSPGAAGTVIGIKSQIGKETELDV

MFDVPIVGGSNERTGPNGLKYTRVFSHQLLNITHAARLRGGDQSESPSDNQPQSSRQRFG

NNRTDQRPQQHHKPGHYKGADSRMDNPKPSHHRAGNSRPENQRSGPPKIQVLQRPKQGDR

QHASDPPGSVGQRLFQNSQNNRRGQNNRHQQNRQCQPKPERNEKAKSVEDQANKGVED

>XRN-2 - L596_g10155.t1

MGVPAFFRWLSRKYPSIIVNATEERPTEVNGVNVPVNSTKPNPNYQEFDNLYLDMNGIIH

PCTHPEDRPAPKTEDEMFALIFEYIDRMFAIVRPRRLLYMAIDGVAPRAKMNQQRSRRFR

SSKEAAEKEEQIRLVRERLEAEGVPVPPEKSPEDKFDSNCITPGTPFMARLADALRYYIN

MRITNDAAWAKIEVILSDANVPGEGEHKIMDYIRHQRAQPNHDPNTVHCLCGADADLIML

GLATHEANFNIIREEFVPNQPRPCELCGQYGHELDECQGLAREEPGPDQCEPMSKSTNFI

FIRLPVLREYLEKELEMPNLPFPFDLERVIDDWVFMCFFVGNDFLPHLPSLEIREGAIDR

LIKLYKDTCFTTGGFLTENGTVAIERAQQILNGLGDVEDQIFQERQRKEVQFKERNKAKR

RRERQQAPAYFPKVGLLAPMSTPQTFSGERTREMARDARAEAMDFTNQQQRMQSIMQPVG

TGNQPQIGQKRKAVDPAESSEEEEVHDEVRLYEQGWRERYYASKFDVSPSDMDFRRKVAE

AYMEGLCWVLRYYYQGCASWDWFYPYHYAPFASDFDRIYDFKPDFSKPTAPFKPLEQLMC

VFPAASRKHIPETWHHLMTEIDSPIIDFYPNDFQIDLNGKKFAWQGVALLPFVDEDRLLG

TLSEVQDKLSEEEKHRNSLGPNRIFIGTQHPAYQLFEEIYNHEGGADVHIDSALAYGMGG

SIAKDSLVVMPGTPYGSAVSHETCHDIESNACISAIYADPAYPEGFVFPAVRLPGCKEVE

KTLKPGDWNQHRNGNYRPQIGFDRHAPRASLDQGAHRGFRNEVRDRSFNNDRRQSDRFDR

RNDRHPYRGQGSPQQHHNRGPRHSYPPPGGHGGHGGGYQHGYPQRGHPHDHWNGGRGGRG

GRGGRH

>VIG-1 - L596_g22586.t1

MEYGINVNNKYGYLSDEEAEDPEVFIKKAIQRKDQKMAEEKKMAEEKAAAEKAAEAATEQ

KRAANKENRRETTERGTRGARGPRGPRGEGFRGLRRDGPKPEGEGTGPRGPRAPRGEGRG

GQNRPRRERPVKEGETEAAVPAGGDEARLNEEGDNRRGGARGGRVFRRGGPRVPGTRLDR

KSGSDQTGVRSIEKKDGHGKGNWGTEQDELAAAEDVNVSSGGEVEREKTEEDLKREAELE

KAARELTLSEFKARMAQKADKPEFNIRQAGEGVDAKNQPKLVPLQRENDEDHVAEEVVVV

RREPKNKRLNIDINFGDENRQRGGGRGGARGGARGAGRPTKERRGKDTHFDFTAESFPAL

GAR

Argonaute proteins

>ALG-1 #1 - L596_g7718.t1

MTTMTGGPQQQQLDQTQQQVISMLDQLSFSEGGGMPPNYGMLGGPLMGPGPLAPGPQQMP

FGPGPPGPQMPQDIYFQQFAPPGMPGQMMMGGGGGFDPRPGSLAPGAPIDPSQTMMPSAM

TPSLVSTQGQGAPGTPSQLAPAIPASTIFQCPRRPNHGVEGRAIVLRANHFSVRMPGGTI

QHYHVEVQPDKCPRRVNREIISTMIRSYTRIFDNIRPVYDGKRNMYTRHPLPVGRDRVDL

EVTLPGDSAVERKFVVGIRWVTTVNLTTLEEAMEGRVRQVPFESVQAMDVILRHLPSLKY

TPVGRSFFSSPTAGGAAPAQGHFQQESKLGGGREVWFGFHQSVRPSQWKMMLNIDVSATA

FYRSMPVIEFIAEVLELPVQALAEKRALSDAQRVKFTKEIRGLKIEITHCGQMRRKYRVC

NVTRRPAQTQTFPLMLESGQTIECTVAKYFYDKYKIQLKYPHLPCLQVGQEQKHTYLPPE

VCNIVPGQRCIKKLTDTQTSTMIKATARSAPEREREISNLVRHAEFNADPYAHEFGIAIN

TAMTEVKGRVLNAPKLVYGGRTRQTALPNQGVWDMRGKQFHTGIEVRIWAIACFAQQQHV

KENDLRTFTSQLQRISADAGMPIIGQPCFCKYAVGVDQVEPMFKYLKQTFAGIQLVCVVL

PGKTPVYAEVKRVGDTVLGLATQCVQAKNVIKTTPQTLSNLCLKMNVKLGGVNSILMPQV

RPRIFNEPVIFLGCDITHPPAGDSRKPSIAAVVGSMDAHPSRYAATVRVQQHRQEIIQEL

TYMVRELLVQFYRNTRFKPTRIIVYRDGVSEGQFYNVLQNELRSMREACMMLERGYQPGI

TFIAVQKRHHTRLFAVDKKDQVGKANNIPPGTTVDVGITHPTEFDFYLCSHAGIQGTSRP

SHYHVLWDDNNLSADELQQLTYQMCHTYVRCTRSVSIPAPAYYAHLVAFRARYHLVDREH

DSGEGSQPSGTSEDTTLSNMARAVQVHPDANQVMYFA

>ALG-1 #2 - L596_g21728.t1

MQQPDEKKPMKPIPLRAMEGSESVQVGRLNVQVNGYTLRIAADTQRTVVHQHEITLFGVF

SRDEGQKDVNLLNMGGGLKDYKKQGRRLIIYAVFDKIVEANPLIFPKNIYQCVYDGGNIL

FCKQQLVDYKLPMESVMESSKFCGSVRDFLGARCEKIKFKITYTGVVPLDTKELNGNSRS

LVQFLDIVTSQQICRSEEQLVFKNRRYDSDSVRDVQASMAKIIKAGSEKTISIVGENSSH

QEALLLIEPKRSPFFMGGNLKDIFDIVQREMGVGNPRLVSELTKLVKGLGVFTLHAKKLR

QFQIKDLTKEPAGRQIITIERSGQATDLTVAQYFQDMYQMAVNPNLPCIACEAIIRGQKQ

VLLYPSEVLSVMPGQRVQTQKQTPKLVEELIRQAQFVPADLMQEVQKERLLFGLENSKYL

AEFGIKLDGKPREAPAKVLPTPAICYGRDTTVQPDQEGRWMIRENQYFKPATQCKWALCV

VENAMDSQVATRFKDSLVRAAAGHGLMIGEPSLHRFQKADPEDLTREFAYLKANKVQFVM

GIFGGDRNCIERNLLKEMEIRFQLITQTIQSKTAFKGTTNKMVIDNILMKTNLKLGGLNH

QVTTSRAYATRFLDAIFPKNRIFIGLDMQSPGSPMLGGINEFTTDPTIVGMCVSVKNAAQ

MRGHYWCQPATFKFIMGIEKALGSVLKMYQAQRENVNDDDFPTDIVVYRGGVSDGDIPMI

VDEEIPQMKAAFANLKIRGRSYCPHLTVLLAQRASERLMPVSSPMDGGGGYNNPKSNGNV

APGTCVSSGIVSPTRSEFILAAHKAIKGTAKPMRYIVLDEYGSQSKRFTIQELENMTNQL

CYTHGIVTSPVSRPGPLYGATDLVKRGRADWKAREYRSRNAAPVGPVDDQFLEGYNKQRL

AWQLEYDGKFWA

>ALG-2 - L596_g16709.t1

MNVFLSTAATQLTIPIDRATDRTGGVSHSPPAILRRIFWDVVDNNRSLFPAFGIVFNDKD

QLWSKRKFNQFEFEAQFGSNNDHTIKLKLVDMFDFQISPTSDQIQAEFLNSLLTQTDRCK

LLSVLCLDCPTAISVCTCAVFLKMRIPIIDYFLSVVSRRPRLTDADYRGLENFHWNPDQI

TALRESLKGLTMVTTYGGTSYYHYKFLDVYRGNASETMFNWRRPENNEEEEITIQEYYFK

KWGIRLRLPHFPLIQAAPLQKNIYLPMELLMISDRPQRFQKPIPEDCMRAALEKATISPR

DRFDLICDMIQQSSINDDNRFMQKFGVYVDTTLMITEARVLLLPILTVRDGHTVIRPDTT

ATWRSREVQSNSRRRAIIAVIINSEVRSSMGNSFFPYFNALMRACRSIGINLVDQQDSFQ

PVIHTYNRDQQEIDVAVXXXXILSFSGFTIPNSVKFFVQRDRLFFIPTEGSRPGADDLGR

GLQMWYALHSLVSVGEKAEAIVNYDRTCAVFLKMRIPIIDYFLSVVSRRPRLTDADYRGL

ENFHWSPDQIAALRESLKGLTMVTTYGGTSYYLYKFLDVYRDNASETMFNWRRPEDNEEE

EITIQEYYFKKWHPTPTALSSDSSRSPAEEHLFADGASDDIRPSAVPGIKQ

>ALG-3 (T22B3.2) - L596_g3457.t1

MIDNIDERVSTPIQVIDIIFPSTSSLTCPLISGATSTESDSASSSSRLSTTDDSGSFVSD

SEEHLTRPSLSPAPSCPQLPKLGAGSRAPSEGSGRFMDGESSESEDEDEAEAQQRRVIAQ

KEAGCGGLLSEDQSKMQIRALMARPGFGTNGRKIPVLANFFEIGIRNKDMIVMQYHVDIT

HPGNRKLDRDENRTVFWKAVEQNPQVFQNRFAIAFDGAHQLYAVQKLRLPHGGSSAEIPI

DIALARDLRSSSKCAINLQLVGPMIVDIGKSKSMNIDERVSTPIQVIDIIFRQSLTCPLI

ANSSNFCAWKSSFYRLPTPNSNDALDLEGGKQMWTGFFSSAHVAQNYRPLLNIDVSHSAF

YKQHIEMVDFMCEVINERASAFTCRATNPGPGAYRGGMKPGGPRGMGGNINDNSPGFLSR

DKLYENFSLSSQELKVLDDAIRGVKIRVTHRPGVVRVYRVNGLQVSADQLTFVGKDSDGA

ENRLTVAKYFELKYAKLKYPRLPCLHVGPPSRNIFFPMEVCRLDSPQKYAKKLSERQTSS

IIRAAAVDAEQREKRIVSLVQQAGFDTDPFLKEFGLKISPQMVETVGRVLRPPAIQYGEN

NRRMDPIVMPKDGAWSMDNQVLYLPAACRSYSLIALVNPREQPNIQNFCQALHQKAQNMG

LMLPQWPDLVKYGRTKECIVQLFKEIAFEYEQTKQQCDLIIVVLQAKNSDLYMTVKECGD

MNYGIMSQCVLMKNVQRPSPATCSNIILKINAKLGGINSRVVPDAVTRKFLIDVPTLIVG

VDVTHPTQSEERQNIPSVAAIVGNVDCYPQTYGAHVKVQRKCRESVVYLVDAVKERLLCF

YRNTRKKPARIIVYRDGVSEGQFGEVLREELIGIRKACMELSSEYRPPITYIIVQKRHHA

RMFCKNPRDSVGKAKNIPPGTIIDTGVVSPEGFDFYLCSHFGIQGTSRPARYHVLWDDSK

FSADELQQITFSICHTYARCARSVSIPAPVYYADLVATRARCHIKRKLGVHETEHFDMDK

FSRKPSTTTTTTTPFDNEIVGARGDCRKGQMPDFTNLKQSTCEAALQDFVNVTEGFKTRM

YFI

>ALG-3 (T22B3.2) - L596_g16708.t1

MTTIAGYGDQSVNLRAAIQTSLKEFYKGTGGLYPSHIIMYRLGCTSSQMTAAAKNELRAV

FSAVKEMAELANQGHFDPTITYLHIERKHQKRFHCGADVGNLPNGFAKANNGNVPAGTIV

DRDVTSQAFYDFYLCSHHGALGTSRPTRYIVFYDDWNLTADQIQAATFCLCFLNSNCTKA

ISLPAPAYYASKGVERGKKYLNTCMLRREDIQPNEPLMMPKSISNSMYII

>CSR-1 - L596_g20174.t1

MIGEVTLADLHAESFGAPLQYPDLPCVEWERQGPARFFALETLIVMPDQRVAFERTDARQ

SAQLQKINTVRPEHRLKNIEDQMRRLHLWGDSQCPVLTGFGISIEQQLLSITAGVRTAPV

IQFGSSTDKLQPGRPKWEMALKKNRYRIPANVKSWALIYRCEDKDVDVVRLFTTKLRSTA

KMRGMNVEVPLAVIRFSPNRQAGEGDDEVLGRHVNEARSAGAEFIVYVSSKCIQDHDLLK

LLERRRLRVTQHISLESVRAVVIENKITTLDNIIHKLNVKNGGFNYTPLIEHVGNSRELE

LASGNVLVIGLDVAHPAPMNASQRRMMHSVDATIRSLEPSSIGIVANVIKNPHAFVGDYH

FQTARREAVEPRILKERVKWIFDLLAQNRPEHQRPKHVVYLRDGVSEGQYTMTIRDELGA

IREAVREIDPKYRPKFALIIVTKRHNKRFFDSSKGVVGNPLPGTVVDHSVVRTDITEFFL

QSHIPILGTVKIPQYDIPVNEGGFFMDELQAFANCLCHSHQIICTPVSLPEPVYAAHEVA

KRGHNNFLEFRRSHPEMVPYVDGNVNIIDCEEVTAKLAYRGTPLEAIRFNA

>C04F12.1 - L596_g11107.t1

MMGSKYPDKLPAHTKGNPKVPLVSNSFEVSLGDKIIHVYDVSIIQDCESRGRKKVIDWST

SSGDSAKRRIKLAVSKEIFKKALEVKKFASKEAALVFDFSKILFSSEKLKDHLCSAIILT

PEDFDVLPSFEGNSKLCRGTYTVRIVPTKAGSHQFRANDLEKALAKNQEDHSLRQFLEVA

TSFKPIQEGTHKFFRGILHDVRTSAPGRRDLQNGPFEIAYGISKGARIIGDMDNPKAALV

MDSKRFAVYSESKETFLEDIQKVLGDKDLKIHLPRITKMFQGVTLGHCFNKAVEVKFSSL

SNVLAEKLTFENSDGQKSFIVDYLEERYENYQCRARRWPVVVDKFPGRSGDVCYYPLDIL

YVKEGQLVPLPLQQEFGITQELLKEVSKPHLRSAEIGRAPKDLELNARNAHLREHGINVK

QAPIKLEGYRAQPPKLGYANGQTAAVDANRANWEAGRYIYPAKVDSFRYFVRQGCMGRDQ

ANLFLNKFLDMCRSKGLDMPKPQIEFIKGPLALKNLLSAEDKAVGKKKSVTFVLFVDSEK

SKTHDALKFYEAKYQILTQQVRSETTFKAGRQTMENIVAKTNEKCFGQNYAIHGDEFIST

KDTLILAYDVWHPTGASAQKRILDIPDDTPSVVGMSFNGGVHADGFIGFYAYQEPLQERV

DVLKSYMQHILRIFKKTRGLLPKNIVVIRDGVSEGQFDMVCQHELASIRAGCRQFANAEK

VGWNPKFMVVTVTKRHDKRFFVQDGHRVLNPPPGTVVDCTVTRPDMTEIFLQPHRPFQGS

AKAAAYSLLVNELEIPKQKSGSDLWLTNFLMKLCYSHQIAPSSISIPEPVKQADEWAKRG

AANLEFLKRENGDKSLDMHNFLKSSSEGSFYDWAALSDALGYHTKRLEGTRANA

>HRDE-1 (WAGO-9) - L596_g11197.t1

MTSTSVNLNSFKIDISETVHRVQQYELVFVLYKSFKPGFTATPSRTRVPLGFKYKYNLDT

FQNGDHWVGGYDIAYGPQNPVKIRMRKEMLFHFFEFFKQQFSHTLAIGDVVPMMIFDCDR

TIYSNQQLKVGRGFSHTWSYIEHLPEKVQDHIDILCGNVSDFCGLSVFLNRVGEVDMTDL

GSEKNPNKGVIQFLETLLWQPLYTQFTDHVIYGKTFFDLAEKNRISVMQDGLFLATGYDI

SVDLIPDYEKDMSKLMPVVRFTPGSSVFYQNYSRMDALLMSLSGTNGRDTLYQLEYDCKH

PSIVEKLSQTVRGATVQFIYDSDQIFEVDHLDPRTPHQITFLLQGRRRISVTDYFMAKYN

IQITSELPCVARKTRYGMSFYPVEVLRVAPNTKAIHKYLPADVKDMIEKGTILLPSAVQQ

RIKEAIGEMKLYSAGIRKHDMARINPFLFVFGIALVDKDRPVRIAAHVSESPLIEYCYRR

GATSTLMKTEQNHPGVWNKSMKFLPPFLKPADLGKPVKIRFVNTFVEEVHLINRFMDKTI

HKLRLKGIDVMKERTISGSERFGKIRDARDIFGRNKDYRTMLKAAEDILTSSGADLFYII

GSTDPMNPTRDIFKLAEITKPSDKRIVTQHIGCKTLCTTVSTGLSKRSPGILESIVMKTN

MKLGGTNYTLREKDRSSGYRYGYPRSTHLIIAFDKVNPQHTDEKGEKLYHPKACAMTYMI

PHTTGLITRGTYWFQTSNETNLTMMISAFEEALKFYHKDSRGYDPEKVVVYWDVSHGEKE

VTQEMDAMMAIVAEQRGESVRGPFLTFITVDQNHNTLLLPTEANVRDRLSLQNVTAGTWI

QESTIGESFTMVSYESDDKMTRAVKYDVKVAETHMHSIQDLTHKLTYLQNSGWRSVSVPA

PLKGATKLAKRAMKSYDIMDQIRGRSGPIEKQLFEERVKEISDWIAVKHLTNYWA

>PRG-1 - L596_g25491.t1

MDARTGQLKSFRAASRQNRGPQTDESLICFAYQNHPRFIVKRTKSISLLKTLILRRFDAL

ASALMRQQKSIIYDFQMVIALKTKF

>T23B3.2 - T23B3.2

MRSRRIYSFNSSIEMSDGEIPIRVVDNRNRDDLLKILLLVVLIIVFPPAAVAVQANECNV

HVWISLFLMLFFIIPSYIHAVWYVFIRKPKELTIA

>WAGO-1 (R06C7.1) #1 - L596_g19943.t1

MSDFENPNVKRPVTRIVDDVEELFERLQLEPGKPVMADKQLPGTTGRKVKLQTNIYGLSL

QKIPVHRYDVNVIARLGEREVLFTKRSKEDAVSTDRKDKCRTAFELAVNTFGEQFFGQER

YALYYDCQSILYSMKPIPALEDKMSHELSLLPHHLTDFPAFTGLDAVVVEIKKVADTFNL

NLGNLGFLNRELIEQDHSLQQFLELITSQHALFTPADHICYGSGTSFLLNHKKYGFLDED

CPDLGEGKYLAVGSHKSVRFVEGPGGRSGAHAAVVVDTKKAAFHACENLIQKAMAIINLT

PQTHCRKNEVDKLRGQLKGLFVHTKHGRRQRLFPIASITEDTAADKQFEDPDGNDVTLQI

YFQQKYGITLRFPHAPLVVVQENKQTNYYPMEVCYVNDNQRVALNQQTPLQIQKMIRACA

TPPAQRVRQNKENMRALELNNSNRYLQAAEVRISNNALILEGRVLQPPDVYYGNGVKAVV

HPDKGSWRLQNKPHFLLAVEIHRWAIYVVGTGNRDILDQPKFENFIRMYMAECRSRGIRI

NDPCEHRILPADPEIIKDRIKAACEGECYFMYFITSDAVTDIHKIMKYAERECGVITQDM

RMSSANDVVAKGKRQTLENVVNKTNIKLGGINYDIRFNSPDLNVLRNDRLFLGFAMSHPA

PQTQHERNKGVAPRSPSVIGFSANMKSSPVDFVGDCVFQEPRRDEKIGVIRGVVNNVVTR

FRDSRGYLPKELIIYRNGSSEGQYPLILKYEVPLIKKALEEVQCDAKVTLIVSQKMHNVR

LMLSQINERDRAPEQNIKPGTVLDVGAVHPVYNEFYLNSHVSLQGSAKTPRYTVLFDENN

YNMDQLEYMTYHLSYGHQIVTLATSLPAPAYIASRYADRGRNLFNASNTNWNVLQDGQLD

YNQITRDLSYGVSDLRDYRVNA

>WAGO-1 (R06C7.1) #2 – L596_g995.t1

MDALKGHCESDETLYKIETALVSKSGTIRFYPQEAKKVLNWAVEPVMVEEDPYKEEDGAF

YSLCHVNIEFLASGVHCIAKLLAPFSNFRRSSATKSSSRRPKTTANVENRSATEPMTNLR

SKIAGPATKTASITVYLTMESVTTKMAHLTMAPKLGAGTGGRPVPLVTNMYQAKMMRAQP

VYRYDVAMEMRFGAKSVSLVKKTIDDMVAIDHKDKCRAAFRIAVRLHDKVLGSPVGLFYD

LQSTLYAIDKIKDAKENTECKEIELCIPSDMLKNNDYFKDRNPDSVTITIKRVEADYQLS

LGDLSFATDASKAHLNADLVQFLEIATTQYAYLKAGDVLTYSSGLIYFSEKRRKLNGGNL

LIDGVQKSIKVIEGSQKGQPQLAVVLDPKKTAFHAKDVAVSDKIYEAGFMEDDGRVHPAK

LETVKNQVKNLFVEVRYGKKPTRFMINGIDKESARVKTFLSSTGETTVESYYLKQYQIEL

QYPLAPLLAANKKVNGERTTIYFPMELCYVCDTQRVKNTQQTSKQISDMIRSCAMLPADR

VKEIKDCAKRLQLNGDAVSGSLRSAGLSVATQLTSVQGRALPPPEIVYKDNKKFSVDPNS

GKWKATGAQKPKFLLGASINRWAMMCVADRPQPRDDALMKNFAEKMVRECQGRGMRVNNP

VFYQAVQGRPDSLESMFRRAKQDNMEFLFFVQDGRLQAHKDIKFFERKYEIITQDLNQQT

CRSVVEQNKFLSLENIVNKTNVKLGGLNYSLIVNAPNTQHLFAKGRLYLGFQVSHPAPLS

DDQKAKGMKPKQPTVVGVAGNITNQPAAFVGDVFFQEPRDDRMADAMETLVRDFALRYKD

AVGVAPAEVIIYRNGASDGQYQTILDVEVQEVIRAALTAAGAGSAKLTYMVVSKLHNVRL

MPAQITGIKAPEQNVKPGTVVDTNIVDPVFAEFYLNSHQTLQGSAKTPKYTVLYDQNNFP

MSYLELMTYVLSYGHQIVGLPTSLPTPVYVAGRYAERGATLLSASRHDSDMCKFSELTEA

LGYGSTKIGKNRLNA

>WAGO-1 (R06C7.1) #3 – L596_g15757.t1

MDTRSQAQGGFDTSIKVVYEMAPKQYAPENKAPVDLVCNAYRLRMPQPSSDYPARVYVYD

VTLTLTRADGSLLTLVKTKMDDYTHYVSKQRCRSALEAFSQKFPGFFRTNEQRLFYDLQS

ILFTRVELPMRNLTEAMSIEASEFSRFGFAEGGQGMQRLNIVVQKTQSGTSIGLTDFSFL

SSDIAEVGQRHDLSQFIEICSSQNAYMNPDQRVTFPGGVSYERGNPGDLVDGSKRVWNGV

RKSVRYLTGAGNPVPAVVLDARKSAFHKEGEMVSDKVYAMGLMNDDGSVFDYNIDNITRQ

LKGLFVMVKHLNNQRTFPILKLDKKTPATYRFTSDDGVEMSVADYMSKKYGTALEYPKSP

LVVVWMKQREVHYPMEMLYVCPNQRVTTNQQTSKLVSEMIKKCAIVPSERIKEIKHQATS

LHLHDGALREVCIEVDPNMLHLQGKAVEAPKLKYGSSALVSVERNTGKWRTSNTKFLHPV

QIKKWAVIVLPTGGKLTGQDSQVPTKFVDLMIKALIIKGVQVGPPAYTGMSSGCQLEEDF

KECARDGYDFMFFIQDSKLQFHKDIKVMERRYEIVTQDLNLNTARNVVQQRKHLSLENII

CKTNVKLGGVNYSIHIDRPEFADFFHDRRLYVGMQMSNSRVFELGDGSDVEKAGKPTIIG

ISANVDRELSSFVGDFMCGPANIEDLSSVVTEIFKYYSEEFLKMRGHFPEEIVVYIGGIS

DGDMPKLLRWHIPAIRHGLNTAKCTAKMTVIFTSKSHNVRLFPKNVTGERAPEQNVKPGT

VVDTGIVHPEFTEFFVTSHQTLQGTAKVPKYTVMIDDNNLSLSYLETMTYVLAFGHQIVA

LPTSLPTPLYVAGRYGDRGGVLYNAFDGKDDLYAVNDQLTFATSKKLCGKRVNA

>WAGO-1 (R06C7.1) #4 – L596_g16574.t1

MPTGEDPVLAEPVASTEAEDNASPRTPHAIHEAGDAIGLERRLEASTSARDGNFYVALGL

PILQGVVGNKKEALAVGVPIELSTNAFGFVLPALPVWQYEIEIEGLLAGSERRVFFTKRS

PDDAFKIRKSQECRRLFQAIKHKYAHSFSEDNETYFYDNQGLLFAIAPLDLSLNEKMDCM

LTAEELAALSPPTYASFSEVIVTIKNVAANPMTVGHVMTYTSQVLENNSRRLQQFLEVLT

SQHMLANPNEFLTYGTRSAYLLDTSAHGLEAGHLTKDKEIKIGCEKSVKLVEGPMRGGQP

DGKAVALIDVKKTPFHIPEGTVLDKARAILNREPKPSDAPRLKKDLAELVVYTKHTSKEH

RYVVENVIADTAVSMTFPWTEEGREVTLSEFFVQKYRQNISFPRTPLLVARFGRERKLIH

LPMELCYVARNQRVTSRQQEVDNISAKMIKACAIAPAERQLQIQETVNALQITSSNPYLR

AARTKITAAPLIVTGHRLEAPKIAYANNEVLSPDARFGFWKPPNAHRRPKFFKPAVINSW

AIVVLPSQAEFLQGDIISREILARFTDLFRSECRDRGMQIGQPVFTEFMKADVQQLRDLI

KSLTRPDPSVCRPPLRYVIFITNGGITFCHQPMKYFERETEIITQDLKMQTVVNVVQQNK

RLTLENIVNKANIKNGGINYVVIRNMPGQRPILKPGRLVIGLAMSYSVRRQAEEISTLPT

AVGWAANITREEGELIGDFLLQESFKKDRVAVIQTIVDRVADAFKHPGGPKEVILYRSGE

EGRFRAILEEDLAVLRATFDNMASKPKLTVIAVQKHHNLRLMPTKINRQDRPSLQNLVPG

TVVDRYVTHPTFTEFYLNSHVAIQGTARTPKYTVLQDDANMSLEELEEMTFGLCFNHQIV

SLPTSLPSPLYIAGRYAERGMTLYRQHQEHEEDQGTSASSPSSGSSGEPHLDVERLGTHI

SYGSSRKLKHLRVNA

>WAGO-1 (R06C7.1) #5 – L596_g20961.t1

MEREGTELRPSTIVRQTQNAAASSAPAAQVSTSAGAPPAAFGIRGPATSAETSFYSQLVN

IPTPPLEEKKQPGTTGREQFKLRTNVFGLSLPKDAQVFRYSVDASGTLQRNDRRIEFAKR

VGDDITYLNRREKCRHVVDQVVAKNPAIFGDRRELFWYDSQSILFSRNQLDIGSEAQFVL

DQSDIGQNTLFEGFAHLKMVIRPAQTNFAVSIGDLEAYIQAELFESDHALQQFLEILTAQ

YAFNTPTEAMSFGTRTAYLLQPEKYGFKPADCADVGDGKFLGVGCDKSVRFIEGPGGAGG

QRAALVVDLKKTAFHKVQSLYEKAREILNNRDPKSTDASRLRMQLKGIVVETKHGSRRQE

FAVDNVVADTPATKKFKDLTGHEVTLQQYFQQKYNITLQHPDSPIVLTDRTKKFAAFPME

VCWVVDGQRVALAQQTPVQIQKMIRQCAVPPADRQRQILGLVQGLQLNSENKYHKAAGVG

ITPTALQVQARLLQNPIIVYGGKSTMRPDEKATWRLARQKPVYLKPVKIDKWAMFVIRGG

NRSDCVDQAILNQFSNMMVQECRARGMTVPDRPTGLSFIGASREEVQETLEKAKTEGNQF

CFFITNNDVTHIHQFMKFQERKLSIVTQDLKMSSAFDVVKKGKRQTLENVVNKTNIKNGG

INYSLRFDDPVFNMDKLLPKDRMVIGLSTTHPKPIPGKKEQDQAPQDKKKQMHEQRTGPP

VPSVVGVAANVLTESIEIVGDCLFQQPNREEKIALLQPVIRSLMLQFMKHRGMPPVEIVV

YRQGTSEGQFRNVMELEYKMVKAAALQQGLNPKITFIVVQKMHNVRLMPTDCKAGDRAPE

QNVKPGTVVDTMVTHPKYNEFFLNSHVALQGSARTPRFTVLYDENRLPMDEIEALSHSLA

FGHQIVNLTTSLPAPLYIANRYAERGHNIFIASQEDYTKSKSSLQSPHSTTIEDNLDFSR

MMNELSYCNSELRDKRVNA

>WAGO-1 (R06C7.1) #6 – L596_g322.t1

MSEELHAASKLKPTEGIASRKVKLTTNHFTLSFKSSKPVYRYDVAMVHYMMTKDGEKSRD

MCKGERDDAAILERQRRCLVLMEAAKKVAHFTPSKSCCVYDNSKTLFSSEKLLENQCAQI

RIEGEHIPDGFKNHPKLKQGYFFIEITPVSTNHKFTIDDLKSGVSDDLLNTDHTLRQFYE

ILTNAYAVTNDSHMVFYGNLYDNDKGESGRKKLKEARNLIFGVNKGARIIEGSSSRLVAA

LVLDSKKSTFFDDANNKGLAGNIRDLLGSHFNAQPHEVVIGDHNRKTVVTYLKDLRVYCR

YQEDRDFVIAGVTKEPIEDLYFEYGSTKTSVMDYHKQAYNTKILYPHWPAVIMQGPRSKN

YFPIEVLGVSKGQRVPISKQTPGQMAETIHDCAARPHIRFAEILKKLDGLNLASNVPNEF

LQSFGVKIDCNPIQVQAHRRLAPKMVYAGNKEVDYDDIKGNWFANNAYILPAKIPKWFVI

TDRIDVSIVRKFAGILKDTMKMKRMTVGEPQFLEMPVAQLDGFLGKMAKELKPEDQSPFI

LFADTNDDSHALLKLYEAKHQILTQHLRARTVIECLEPRKRLTVGNICNKLNCKNYGLVY

AVNPQDHAKTMYLSKGDVMVVGYDVSHPEPQPAHERRLGIPPTTPSVVGFSFNGGVHPGM

FIGDYQFCAPRQERVDILEERIQWMLRVFTTNRKTLPSRIVIVRDGVSEGQMPMVLQHEL

ESIRRGVKKLKAGYNPKFLLVTTTKRHAKRFFAETERGIDNPMPLSVIDHTVVRPDVTEF

FMQAHKAIKGTAKMPAYTLLLNELGMTLDQIQSFMMGLCFEHQIVNSPISIPEPVYQSDE

WAKRGHSNILAFFRLMDCPDPQKPSQKMIQRYLKASENPTTGQPEVEGYDWVRISKMLCY

RGRRLEKTRANA

>WAGO-1 (R06C7.1) #7 – L596_g9675.t1

MLEIRIPPIVDDQKRRSETELNTVNVRTNVYEFELPTGTSEIYHYEVAVIGKLKSGRTVD

LTARTTNDVLTLERRDSCRKVLGIVSERYPAVFDHNLVFYDQARLLFTSKNVEINTAAGI

YSVILGCQDHLKGDEMFAAFTSVEFRLARVSVVEVGNVQKYLNKNLRLVNHSLEQYLNVL

TSQHVANKTITFGNESVYLEQDDQCGVRNNVDLGSGKLLKTGFAKASRLIEGLTEQPAAA

LVVDLKKAAFHVQQTVLDKARLILQADRKRCGGSQPRIEDTEDLSRELVGLVVETKHGSR

VRKYKIAGFDKSTPASRSFEKDGRNVLIADYFAQQYKVKVENLDTPLVIVRGYGRDFHLP

MELCYVKDQRVGLKQQTPDQIKDMIKQCAVKPVVRIAEIEKIVKGLRLIDERKDILGSKV

GIKDKPLKVEGHLLPPPLIVYADKDTCNQRVPPSREPASNGTWSSLTANTTPAAFFKPAK

IEKWAVIAIHTEDNGDPHDERNKRILAQNTLETNILHQFATVFTEECRNRGMTLPEAEHV

KFLDDTSTTVRDFIHQAKLSFVLFVCNNAITHIHQSMKCYERKFETVTQDVRMATVNDIM

TKRRFQTLENIVAKTNVKNGGLNYNVEMPSKNGVRELMPKGRLVIGLTVRVVRVPKSPKE

IEEDKKKAEEREKQHKKARDSKFARKESTTAKLKPIMTVGYAANFTDVPTEFIGDHLYQE

YRETGDILGMQIIFERVVLEFKRARGMFPTEIVIYRECTENSDFIGQLQLEQMLLKSAIK

STASFRPGFEPKLTLIAVQKRHHVRLMPIAMRREAKAPEQNLQPGTVVDRMITHPEFTEF

YLNSHTTLQGTARIPRYTVLKDENNLSMHEVQLMTYGLSYAHQIVNSPTSLPTPVYVAVT

CAERGMNLAKHNFRAMNIKFNANDDYSKLEDQHVDISHLNEQLSFGNCKLSSIRNNA

>WAGO-1 (R06C7.1) #8 – L596_g18735.t1

METLTKAVANMMPPVNLASKREDAKPNAQERALPLQTNMFSLSMRDEVPVFMYSVDVFMK

VRSKAISLVKHSRDDYIVIDRKNKCRAAFRFVVRANPAVFGNPGKIYYDLQAQLFTLEKL

NMDNEDEGLELVIDGADARRSTDFAEIPLDGIVVQIKRAGPKFDLALGELQLKFVAQEPK

SHELLQFLEVATSQYAFLTPSDFVTYPAGLSFIKQKDPKAGTELEGGKLLLDGAQKSVRL

IEGEKKTDGGGKLAVIVDAKKTAFHKTYQKVIEKVNDFGFLQSDGTVHRMRIPDLAKLLK

HIYVESRYRKRTQRFLINDVNPDNARNKMFTRNGVMISVEQYYREVYNITLRYPLAPLIV

SKPMKSKDSEEKMVCLFPMEVLFVCPDQRVKINQQTPRQISDMIKRCAMQPDVRVKETKN

YAEKLQLNGPASQACLNYAGVSIANEVVKVSGRKLVAPEIQYKDKKIAVDSNTGTWRSSG

REKPKFLVGSSLKKWAVYLLGVRQPLPDENLGRKFVMRMLEEARARGMQWESPTAVKGVL

GHVDNIEKLFKAAQKEGLEFLFFIGDQKVQVHTELKFFERKYEVITQDLDLKTCRNVVEQ

GKFLTMENVIAKSNMKLGGVNYSIQVNDPTIKQFFKPGRLILGFQVSHAAPLKPDEIAAG

VKPRVPTVVGVAGNVSKGDPACFVGDFFYQTPREDRMLEAMDTLIADFAQRYKRATGKDV

AELIIYRNGASEAQYTDLVRDEIPSIRDALQSAGFRGVKLTVMVVTKLHNVRLMPVAIKT

GAKASEQNIASGTVVDKGITHPKRAEFYLNGHVTLQGSAKTPKYTVLTDDQGFTLQQLER

MTYALSYGHQIVSLPTSLPSPAYIAGRYAERGALIYQGFNHTVQHDADLQTLNGTLGYGK

SKLGDKRVNA

>WAGO-1 (R06C7.1) #9 – L596_g18947.t1

MSNPSHNGGRRPGANGNGNGSQPGGLEMRAATHVRQIRSGASSGSMSSEVRYYQELTQAG

QQLHLEEKKPPGTLCREELIVHTNVYGVGLPDVQVYRYDVDACGFLERNDRRVEFAKRAV

DDVADTTRRNKCRAVVQLVCQNYANIFGNHREYFWYDSQSILFSKNMLDGIPEREKKEFV

LTQEMLAGNPMFRGFKNVRLNIQRVSNSFAINIGDLTQYISADLEENDHSLQQFLEILTS

QYAFNTPSEAICFGSRAAYLMQPDKHGFKAQDCADVGDGKYLGVGCSKSVRFIEGPRGDQ

RAGLVVDLKKTAFHYEQSLLAKSRAILNREPRGNDAGRLRQQLKGLVVETRHAQKPVRFT

IEQVSDETPRNKKFMLQPDNREVTLLQYFQEKYNITLESPDSPIIVGDRLTKFAVFPLEA

CFVVDGQRVSMDQQTPTQIQKMIRACAVPPADRQRQILSLVQGLQLTSSNVYHQAAGITI

TEKPLLVRGRILPNPKIIYANNVVVSPDAQKATWRLDRQKPHYLIPAKIEKWAMYSIRAG

GRSDVLDQPTLLRFAQTMVTECRMRGMQLGNPGEVSFIGCAQEEITATMEQAKKDGCTFC

FFITNNDVTHIHQHMKLLERQTGVITQDLKMSSAVDVIQKNKRQTLENIINKTNMKNGGL

NYTIRCEGLSNEQLLPSQRLIIGISTTHPKVAQTALEDRDRADKNEDPLTKKKPHHEKHH

RSEHITPSVVGFAANFKKDPIEFIGDCLYQYPQRDEKIGVMQPLLRAVINEFSNNRGVPP

NEIIVYRNGINSISTMELEFLMVKAVARCQGITPKVTMIACQKMHNMRLMPAKINPRDRA

PDQNLKPGAVVDSNVTHPKFNEFYLNSHVCLQGSARTPRYTVLHDEGQFSMDELQALTYN

LAFGHQIVNLTTSLPTPVYVAAAYADRGHNIYGVSQKDYTKSRTSDLNSSTLEGNLDFNR

IMMDLSYSNSELRSKRVNA

>WAGO-1 (R06C7.1) #10 – L596_g1747.t1

MASVDVELREKTKIRQEADRMFKEDYGIDVGSTEMPAKMMKPAAQNTMSLVTNLFPVKTH

QQMPIYRWDVDITIAGKNGKTFSLTKKSDSDAVAVNRKLKTRSIFKRLVAAHPEKFGKLE

ENYYDLESMLFTLKDLSTDQEAQDFVVDGLDAKIIPGIASANVILKKVTDRYQVDLTDFR

HLTRDVANVDLSHKQFLEVVTSQYPLMAEDYVCYPGGVSFSVKEKTTPLAEGKYLGHGVQ

KSVRYIENDGRSPCAAVALDLKKTVFHSEINALEYLKMQNTYVKQLIESKQRAKDNDMTL

KTACQRIKGLRAYTQHTKRQRVVTMDRVGPETANEARFQLQDGEEITVAAYFHRTYGAPL

LFPNAPVAVERKPGSKDVNYHPLELLTIAENQRVTGELPQAVISDVIKQAAVVPLDRQQQ

IMRAHRDLKISTNDYVDNVGTSIAPQMMQVKGRLLMTPKVVYGRNTQIEAREGKWRAERK

TFLKPAGAARWTCMMLTNNRLGEQMMHNFLNKYVAVCRRNGMQMADPIEPFVVDWRRTDL

QTEIDAFMKDCTQQYKLEFVLCIQDNNMHEHKYLKFLERQYGLITQDICTRTVERCIGNA

SATIDNIVQKTNVKLGGLNYGLEYKSPDGRHDVLSATTMFVGLGMSHSKPPKPDATGQEP

SRPASVIGFAANVLAQSFAFVGDYYFQKADRDEKIFAIVPIVTRLLEQWCEHHGGQMPKN

VVFFRNGGSEGQFQMILKYEVPLIKFAIEEFAKKSAVQQQFETKFCLLVANKRHNVRFFK

SNITSGGRAAQQNLQPGVVVDSGVTHPVFSEFYVNSHTTLQGTGKTPRYTVLHNDCGLTL

NQLEHFVFALSHGHQIVNLTTSLPTPAYIANDYAERGMAVLGQFLRASGAPVDEYETFNS

KLTFAAMHPGSKFHHCRVNA

>WAGO-1 (R06C7.1) #11 – L596_g1696.t1

MERFKKLPPTTPLKARQVTLVTNHFRLNPTGRTVFRYDVAMSHTRMVKGTEKIRDLCKGD

RDDAAILERNRRCLALMDAAFAAAPFASTSAAVIYDNSKTLIAAEELDMRQCACIRLEGG

TIPPGFINHPRFKEGYFTIQITPTSSNHRLVINDLEAALAGDEANPDRSLRQFLEILTNQ

ETLKKNSFMAFYGNLYSREAADQRKLREARILKSGMSKGARIIGSSSDLVAALVLDSKKA

TFFDDTNKNGLAGNIRELLNRAPNDAPSRVHINDFDRPDIVKYIKDLRVYCLNKPDNTFQ

ISGLTREPLRNVFFDMGGEQLSVLEYHQRDGARLAYSHWPAVIVQSPRGRNYFPIEVLGV

CEGQRVPISKQTPGQMKVVVNDCAVLPNVRFAEIHQMLNALNLASTTPNRYLQAFGVTID

VRPMKITAYRRQAPKIVYGGNIKVQYDDVKGSWFSSGPYVLPAKIPKWFVVYDGIDQRTV

QQFVGVLQQAMKDKRMEVAQPKYMEMKVAGMDAFLSSIVKSLKPGERSPFVLFTDANEDS

HAFLKLQEAKHQIVTQHLRTKTVRECIEPRKKLTVGNILNKLNCKNFGLNHLVAPDHEKN

YLQKADVMVLGYDVSHPEPQSPQDRRLGIPPSTPSVVGFSFNGGQNPEMFIGDYQFCPPR

QERVDILESRIQWMLKVFNDHRKKLPERIVVVRDGVSEGQLDMVLQHEMASIRRGAAMIK

EGYKPKFLLVVATKRHQKRFFIDKSNGEVDNSMPLTVIDHTVVRPDVTEFFMQSHKAIKG

TAKVPAYTVLQNELGMSLDEIQAFLMGLCFEHQIVSSPISIPEPVYQADQWASRGHSNVL

AFFRVMDIEDPEDPSKKMINRYLKPVEDPLPGRPKFEYDWTRISKLLCYRGRRLEKTRAN

A

>WAGO-1 (R06C7.1) #12 – L596_g18749.t1

MPLITVEMSLFVAEGSVVIFFNAIVLLIIVTDSTLRESTELMFIGGLCFADMTDGIAYFY

AGIHRLCNILSRTDDVMISRLECFQKPFMFFFFYGYQLPAMMIFIVALDRFVAVFAPMWQ

RKVERSSKLMVMVAIFLWVTLTYVINVFLFHSYSTGYTTAQCFAHDVFLSQLWDFIIVQR

SILILLCVLLYVPILIKTRRIFQSNDKSVQSNSFNVTIGLTMTCSVLLLFIPDVIIYFDL

IMDFHLILYLLGLNKCVANVFIYTLRQKEIRKKIELICRRVFCNLKGPFDLELSKRRQRS

NITRTLGIAKRCQSLSAVAWSVVLSSAVPQETWHVISRPPLGEARFDGRIDKGFIAPLLY

FYVSKMSAGPSNLQPDVELRPATKIRHAAEIKFKEMGIEVGIPEMPPKIPVPPNGNVKLT

SNLYAVRTSSQIPIYRYDLDITISQRNGKQLALTKKSDSDAVSIDRKLKTKIIFFKMVQT

YPELFSGVHHCIYDLESTLFTLNDIMKDDPEAEPKTLVIEGLDGAKFQGTTSATVILKKC

HDRFEVDLTNFSHLELNIESTDLSHKQFLELVTSQIPMMSDSFVTFPGGVSFSMNASTTE

LPEGKYLGHGVQKSVRYVENPQTRKPMAAIALDLKKTAFHAVLTGLDYLSQIVDDRPLHN

GQKLPNSVFGSFGALKKMKGLRCCLQYGNRYREVVIHEISNKTAQEHYFDREGQQITVEN

YFYQMYNLRLKCPTAPLAGEKKPGQKALCYYPLELIRVLDNQRVTGELPQKVIRDVIKHA

AVVPALRIRQIQDSCADLGLFGNDYLQNVDTVIDSRPIGVEGRQLKNPKMVFGKNTSVES

RNGQWPNRGAMRPPFFIPSTIRKWSVLMISNLQNGFASETMHTFVSSFINECRARGMTLP

APSDPWALNAREELKPQMEGFFSECPGLGIEFVLVLQDDCFHEHKFLKFIERKYNVISQD

VNMKTVQKCLQRAAATLENIVQKTNIKLGGLNYSIEMTNPAGTSNVFQPDTMYVGLAMSH

SKPPKPDSTGREPPKPASVVGFAANVLPQNFAFVGDYYFQAADRDEKIDSIVPIMHRILD

MWCQHHEGQMPKNILFFRNGASEGQYKSILKFEVPLIKHALEKFRNNCGVEQMTPETKFS

LVVATKRHNVRIFKANIQAGRPNEQNLPPGIAVDRSIVHPMFAEFFVNSHTTLQGTGKTP

RYTIMHNGADFKIGQLEHIVFGLAHGHQIVNLCTSLPTPSYIAGDYADRGMVVLHEFLKT

TRAEADQYDTFNEQLTYAALQPESRFHYCRVNA

>WAGO-1 (R06C7.1) #13 – L596_g18732.t1

MLGKWRTVRVEFEAAEELDFRWEFDHMVAAIFEALQPHCRCLLGFGTRSTFQINTVYPKE

LQSPAILFPDDPNGLCKRNLLVQVSLFLLLKSFQLHDFSSSQLSGRFLLLFRTCGESECE

RKRPRFATFGLPNLRSSANVQKLRYANSIAKTERAANRRSNPLQTNSSPGPARKTAPHRN

PAANPKTCFAIGVRGQVADTVDRSFVPRGPLLTVAPPALSPSALPVFSPIRLFTPPHPAA

YFRQSTMDLHTKLAELSVADNAMKPTPATEFKETVEVVSSYFEISIGQNALAYRYEVDIL

AISQRGQEKNLTRGPADDGAASLRRQLCHEVFNAALKKSNGFGTKQGVRLPVVYDCRATL

FLPAPLKMEDEVVVILDKDADFAEMSKETLFTLDPTDVIRVRIAPTTQNAFEMDLRAELM

KADFCEPDCEFARDRSFRTFLEMLTSVNAVRSGSHTQLGVGNFFDNDPSMIVDIGDAKCL

RPGLSKGVRVVEKDDRPYPALVVDAKSSCFFKAQNLAQSIMELGRQKGRPDDMWKTARFL

FKDVRVISAPVDKKGRVKSFPIRAITKMPATELNVKVKGFNGSLADYYARVLKIKLQHPN

FMCVEADVPGPKKEFFPVEVLFVSPNQRVPIEKTEANQSSIVLKANAIKPDRRIKNIKDQ

MSRMSLFANTPVMEAFKVFVCPDFIRLTAGVRVAPQISMGDRDVKIDQKKANWAKEANGA

NYKQESFSLDSWAVLYANTEPGLIRQFVQRYVAAAQRRGFTVREPTILKFNQDFERTFSE

CIDNDIRFLMLIDPKYVKTHESLKLFERLCHVLTQHVSLERVFDVVQKNSRMTLDNILSK

THMKLGGLNYVPIIENVGSRFALDSGEVLVIGYDVAHPTAMSPQERRLVRSLNLDVKSLE

PSVVGITANCSFNPHEFIGDYHYQTARKESIDVSILERRMVWIMRLLEKNRPDQCRPKHV

VILRDGVSEGQYDMARNEEMAALRDGLKLVDPEYNPTFTLVIATKRHNKRFFGQDGRNYV

NTDPGTVIDKTVVRKDVPEFFLQSHYPLQGTVKIPQYNKLHDEANFSMDELQAFVNCLCH

THQIVNLAVSIPEPIYQADELAKRGRNNYAELRRRFTSEVPRMNEAGVIDCNALTKILSY

WDSPLEAVRFTA

>WAGO-1 (R06C7.1) #14 – L596_g20920.t1

MEPSSQPPPTPSLPENMAPKIVGPRDIYERPVPIASNLFPLEMKADVPIFMYHVQIHMKI

GAHEINLVKRHTDDYMHIDHKDKCRAAFRFAVRSSPATFGDPKGLFYDLQGQLYSDHKLK

DVLGHDLIVREEIGIPGEEAGRAPEFKNLGVEYLRVEVEPTRENRPTMILGEMMATRAKM

QESVSRELTQFLEVATSQYAFLTPSKMVTYPSGVSYFKPTTAEPVQVFPAAKELLDGVHK

VVKLIDGTKRGEMAVVVDPKKAVFHKSNITVIDKTVEMGFLDRGSGTVLRETIPELAKKL

KNLYVETRYGKKPIRFAVHDVVLDTARTSRFNKNGDMTSVEEHFKDEYNVILKHPHAPLV

VSTPLKKNPESLQLLYFPMELLFVCPNQRVLFNQQTAKEAFAIKKASTILPENRLKEVVN

SASKLRINGASVQGCFKKAKIEVGNAPLTVEGRSLVPPHIEYRAQQVQVDAVSGRWRSSS

RRGKPQYLVGGKIERWAMYVLSQAPSQAEEELGKNLLAKMEDEFQARGMQIAQAKFLATV

KASPEYMKKIFDRAKAHQLEFLFFIQDTKICLHKEMKYYERKYEIISQDLNMETAKAVTE

EGKYHALDSLIAKLNVKVGGTNYGLVGPSIPDLFKRGKLYIGFHASFSANPADAEDPTVI

GSSANVTQTSAAFVGDMFFQEKNDVDMNTAMAKATLKYVLRYKNVHGHAPSEVVIYRSGS

SEGQFGQILRDEVPALRNALQNAEADEAKLTLLMVNKQHSVRLMPSVIMPGSRAIEQNIK

PGTVVDTKITHPRFAEFYLNSHQVLHGTAKTPKYTIIVDDSAHQIEYLERVTYALSYGHQ

IVDNPTNLPSPLYIAGEYADRGLTLIKAKRKLGDTVDVKDLEKDLPYMASQVLADKRVNA

>WAGO-1 (R06C7.1) #15 – L596_g16096.t1

MSNNERVKTIQNGMETISISATMPVKKKIANAPGAPLQVATNIYKVGLSQVPIFRYDVDI

TLKLPNGKDVKVVKKDRADHVIIQNKNKACKTFQKAVQKFPNVFGNVELFYDCQSILFSL

RKLNISDKGQEFTLSPNDLPEVYGHVDSVNFIVKSVREKYQLTLNDLGFLSTQEVNGIKH

DLAQFIEVATSQNALFNGKHSVYDGGVSYLMSTGEAVRNEPSKQLITGVQKSVRFIEGQG

KPEATLVLDLAKTVFHKGGQNLYEKAISSVRNWPRNDQVDRREIKSLSQQFKGLSIKTEH

QKTKEYELAGLSEFSAHTHKFDHNGKQMSVAEYFKKQYNKNLMHPNAPLAIAKNFFMGKR

NTMMLPLEICTVLPNQKVSKQQETPLQTAAVIRYCAVLPSDRKEQIYTQVKELGFWGNKN

LPITVDQQPIVVTGRQLPQPSIVFGGNQTVSVNPANGKWQATKGVNRNARLPFALPGNPM

PWAVVLVGQGTQDTAKSFALAVKTECESRGLKMGDPSAVIQANYEDIEINARNNTFENLT

KMTPRVKFCLVIENERSPAYIHALIKKNEQNWRIITQVTDSATVQQFNFALGPAQGYGKG

QTLENICLKTNVKFGGLNHFIKPPQGHEKVFASDRMYVGLGISHASPISDAQRARNVKPS

PSVIGISCNYLAHPQALAGTFVFQEPRENKMVESLKKVFFDLATKFKNIRKVIPKEVVIY

RVGASEGQYATILEQEVPQIRAGLKEAGCNAKLVLIVPSRTHNVRLFPQTIKKEDKASFQ

NLKPGVVVDSVIVHPKFPEFFLNSHCTLQGTANTPRYNVLVDDLQAPISDHEMATYMWSF

GHEIVGSPTSLPSPAYIADKYAERGRVLAVEARRKDEKFLNEEGEVDFTEMTAKLSFAGT

PLANFRVNA

>WAGO-1 (R06C7.1) #16 – L596_g21757.t1

MSEISVDELSSYISLESMSIGSSSAYHKKAPVPDSSLHHPVELLSSYLEIGIQEGSKAYR

YDVEIEASKSGSLTRGPADDGHGAMRRKVCYDLLYAALAKSKGFGTGQDYHMPLVYNRQN

HVFFAQPLPEDFITIDLDDNADFTGITNYLYYMTGATESIQVKISKPQSEEHVLDLYESI

FDTAPLDCDGNLEISSRDRSFRTFLELLTSGPASFTGSHVACGTSFYEATNGKDMKDGKT

MRAGLKKGVCVIERDGILYPALVVDSKTGAFFKEQNLLKSMKEMNNGEVPRSAAEPMWQK

ARKLYKDVRVLVVSNVYKNSTQRRLTFPIQDFTREPASRQMMNMKGFRGTVEQYFRQKHN

LSLLHPHLPCAIHASNSKIPVPTFFPIELLFVCGDQKVPLEKSDRFHSETLLRENAVDPK

LRKERTEVQLKKLGLWREKREKMSVDDKKDLSEDGTNLLTAFGVSIINEFIPIRAGVRVA

PTIEVANSEKISIVQKTANWEKKLTNKRYFSSVEITNWAVICSQISAENPMIVRFLKQMV

NVSKRRGIRMDSPMKYSLKSGSREDFDDIFRHIAESGRHFVMYFSPLKEKQHDLVKWMEH

HYSVVTQHVCLERIENVVTGRIQILDNIIHKANMKLGGLNVIPRIEKLGQRMEIESGDFL

VIAYDVCHPAPMTSRERVLMRSMTSFDPSIRSLDPSVVGIVANCVAHPHAFVGDYHYQAS

RKESVDGRILVDRVKWIFELLAERRPNASRPKHIIILRDGVSEGQYKMAEHEELSAIRRA

VAMIDPNYHPTFTLVIATKRHNKRFYDKNEGIAVNTEPGTIIDKDVVRGDVTEFFLQSHF

PLKGTVKMPQYAILCDEADFSQDEIQAFVNCLCHSHQIVASAVSIPEPIYAADELAKRGS

NNFAEHVKIHGRKSLRKDPMNPNLIDFEALTHELAYWKTNLEAIRFNA

>WAGO-1 (R06C7.1) #17 – L596_g18751.t1

MSDQPSSSHQLDVQLRPATIIRQDAEKMFKELGIEVPEPIMPARIPVPPNGNVRLVSNFY

PAHVNGQVPIHRYDVEMTIARRNGSQLALTKKSTSDAVSIDRKAKTKAIFSKMVATHPEL

FTNMYSCIYDLESMLFTLKEIEEVNLIIDGLDGEMFQGTTSATVNLKKCHDRYELDLTNY

GFLQADVGSVDLSHKQFLELITSQIPMMSEEFVCFPGGISFGYHVDATPLSDAKNLYHGV

QKSIRYVENPATKKPMAAVAVDLRKTTFHQSINAFQYMCAQVQVDRNGCLTNASSLTKVS

KAMKGIRCKLTYGRRYREMVIHAVVNKSPRSTFFKRGEQDISVEQYFYEVYQITLRYPNG

LMAAEKKPGQRELCYFPMELLEILDNQRVSGELPQAVVSAVIKQAAVLPAQRRQEIQQAC

NDIGLFDNEYLRNIQTTVEPRPIEIEGRLITNPKLIYGNNVLVDSRKGQWPNRGANKPKF

FLPAVVRKWTFLMISEQRNGQATGTMNGFLKEFIRECQMRGMTLPHPSSPYVLDTNRNVE

DQLEEFMRDCSEGDYEFVFVLQDGIFKFHKLLKYLERKYQVITQDLKTQTGQRCLQRAAA

TLENIVQKTNMKLGGLNYSLQMTNPGGRKSVFDESTMFVGLSMTHGKPLKPDSTGELPPR

PASVVGFAANTLPQQFAFIGDYYFQAADRDEKIDSIVPIMTILLTKWSKHHDGQMPMNVV

IFRNGASEGQYKNVLRFEIPLVKYALEKFREAADVEQIHPETKLCMLVSNKHHSTRIFKT

NVPVQGRAPEQNLEPGIAVDRAIVNPVFQEFYINSHTTLQGTGRVPRYAILNNDAGYALG

HLEHIVFGLAHGHQIVNMTTSLPTPAYIASDYGDRGMVILTQFLREFEKLKGYEHPVDAY

QEFNQSLTYASLDPDCKFNLCRVNA

>WAGO-1 (R06C7.1) #18 – L596_g4755.t1

MLSVEEQAIHPGRCGKRLQVNSNVLNVRLPASRIYHYHIDVVGHKRCGPKIVLSRSFFND

SLGHERRTVLVNLFNWLAYFNEERIFAQPRNFLFYDAKSNLYTRKKLNCKCGEVVTISTA

QLTEAQIEGLEEFLCVRVKFTEADPFEIVLNEPEEFRDLGGPLQNFLETLFMQHSLFKNY

ESVVMNPLIQVAADYEARGIPEVEASTLVKDGSRIFPAIRTKLMNMEGPQALTMEGLHEA

DHACLCFRWEPFAVHSSVTLDQKARRALEKEGFESFFPFELDALNRELCGIRVFTELSGT

KKYFIVDHVSERTAWTAVDGQTLKDFLKTEYEVTLKKPDLFLIVEKRGSLDIYHAMELCT

VAPFQRPHKKLNVPPRFKWDCHQMKNKASPSKHTDHAKRLRESVGLVNENVFLQGSGVTL

ATEPMEVTARILESPMLRVKREGLFAVKSKGEWRYPPTTHFVHSAKIERWGVCLIFQERM

WCSDRYQAHIRLNNLMKVISDRAGGHGMRMAERFQEPYNVPIREGSSLSDQIEQVRLCFE

RCKETFDPQFLFFVIQEGIHGLRDCIEAFERKYQIAALDVNNNEALKMALAHCSSRQKSP

MAQVLTSTNIEAEKALRTLMAKINTKMGGLNFEVVPNHANRLLLQDGYLFIGIHAAFAWY

EQQRATVVGYAANLRYPMAFSGDARVRKFDESLPEFLAKIVKRCCIQFNKVRGVDPQHVI

IYQSQPSKDLCQLIVDRTAILLGEIGISTELTYVFVDEHHDIRLTNTHLNNTSVIEEQNL

LPGTVIDTHLVRKGASEFFLNSHIGLVGLSEVPKYTAHDFRSGLDGDNLQSLTYTLCYAV

QSLNAPVSVPAPLYVAKNKARHGASCLKLTGKVGARDFVN

>WAGO-1 (R06C7.1) #19 – L596_g20173.t1

MADEVIGRMRQLSICDDPAYALAPKLQRADKKSFVQHVDIVTSYVRINIVGPAKSYRYEV

TIEALAEGREPNILTKGPADDGHAVMRRTVCYFLLYAALDKSGAFGTGLGHELPLVYNGQ

TLVYFAKPLDRDVIQVTLDQHDDFANVPEELLYMVSGNDTVRISLQKALVDSEMDLQTQM

FETGQADVENLSTHDRSFRTFLEMATQQAAHYAQSYTSIGNAFFEKDPSRTRSLGDGKII

RPGLTKGVRLIERDGVVYPALVVDARSAAFYKEQNLFLSVKECMDSGDPNMSMNDKWDRI

DNLFRGMSIFLAT

>WAGO-10 (T22H9.3) #1 - L596_g19923.t1

MSFRIPKKRKPEDEGAGGTIPPKQNYSSSSSYASASSSSRQTSNGGKNASVYMNGFELQI

GRSVEIHKYEIKLFGVFRKRNGDEDRKDLTQGSKEAKDDISIQKRRRGCWEIFRAVVREN

NSLFGDRNHRFVYDCGLIFYSIDAIFPETETKTGTFDVAILSRECQDYYGQAMKMIEYSI

KKVRDGTFTLGVAPEDKDDRSMQQFLEVLTSQGVYAEGLDNLIFRNHRYDVKYDTPVLRN

KPEYPFCVRHGSSKSVFVAEIENSNKALQTILQLEKKTSPFFPRMNVLQFVDQSKSDIGE

RIVRGLFVTTTHLKKQKVFRVADFSKMNCNDITFKMRDRQNEDEFREISVTEYYQEAHKF

STRAGHRPCVVERKKRFNGEKEENNYPMDCLEIMDGQRIMDKKQSGDITAYLISEARVLP

RQMGDEIKEELNHRVLNQEAEKYLEAFGVRISRDLLRSDAKILQAPKIAYGDKEKYLTTD

NGQKNAWKIDDALKFYRPGKISDAGGENEQNRWIFAVLNEFQSERDHAAAKRFLGKLQDR

AKLRGMLMLNPEVRCLDVRDPDPDTVNKKLSELCQYAKANKVKFIMFIQYERKDMSRDTM

KQLETKFKLTTQQITMSTVNKGAGDKGDRMVLDNILNKTNEKLGGINCIMKPSPQIAQWF

SGNVMYMGLDISHPGLGGNALSSVTPTAVGMTFTKNRDEVQGRYWFQEAREHMLRSLKKQ

IVFAIEEFNRCSRRYPDKIVVYRGGVSEGEYDKVKTDEVEQFMEAFREINFPGKRKPALI

IVIVQRNSGYRLIPTQDNDFRGNDAIVQNVLPGTCAEVIGEGGRKEFILVPHQAIQGTAK

PSKYVLIYDEAKCITLSELETLTNTLCYSHGIVTSPVSCPSILYQAGDLAKRAINNYRIH

SSRRDFGAMPPIEEVDKRNEYFDQMCDMLQVTLDTRFWA

>WAGO-10 (T22H9.3) #2 - L596_g15911.t1

MLPNRAPQGNELQRNRKVELQVNGYRMRINESKVYMHEIKMEVAFNTLKGLRNVDLLARP

ANDVIRQKRRRLIWSIFHATRIKHPQQFLYNEYEYVYDCGGALFALHQIGDGNRIEFVMK

MADFPEEAKGQLHRAEHVILRLAFTRILDLRQSNLYDGGEAGEQRCRFVQQFLDILTSQY

VLGSDQHLVFQNSRYSADPREDIEAHLSKVIKKGASKTINIVGDQKNQEALLFVEPRRSP

FMADKKVLDIVEEVRRELGGRSSNAQLKKKLEDLLKGIVVETIHQKDAVQFPVKGFSAEP

AGVLSFTMDAGESTTVADYFKRRYHLHVDRDMLCVVCERRQQKFYFPCEVLVVLPGQRVH

CSRQTPKLVEQLIKESQQLPSRMKEEVNHEREVYGFHELNQQLKAFNVTVDTELCTAVGK

VLPPPVIQYEQRTVSADVDRSGQKITGRQWKVSGQRFVRPAPTPEKWVLCVFENALESES

TRTFARAYVNAARSHGLILGEPIIERLQEVNQSTIYARGQAYKSNDVKFILFIFGGDRKN

FERDIMKESETLFNYTTQAVNTKTAMKAISDRGAFMVLDNLVMKTNLKLGGINHELANAQ

DFPQGYLEKVLFKAGRVFIGLDLQSPGMLGGADEFTLDPTVVGMTFTLGSPADMRGTYWY

QPAKKKYISRLKDAIEDVLMVYQESVGSLPNDIVIYRAGVSEGDILMVVSEEIPAIKDYL

TTLDNPDGCPYRPHLTVMVAQKNSTMRLMPLVVHQTGRAQDENVEPGTSVSTNIVSARHT

EFVLAAQQALKGTARPTRYIVVHEEEGQFTVEQLENMTHQLCYLHGIVALPVSRPSPLYS

ATDLVKRGRANWKVRMERKEGSHTPLGPVNDTFFDPINEERLRFLPVTLPAKFWA

>WAGO-11 (Y49F6A.1) #1 - L596_g17524.t1

MNKSSKIAAMPSFKKKRPAENDDSPAPKRPAEKARRSQREEAPRRSHGGSKILMNGFRVE

IEKAMTIHKYYIQLNGIFKKRGGEEIARDLTEGVHRDDVSMQRKRRGCWDVFRQIVKENE

SLFGGNTHKFVYDCGRLFCSMEEIFPKSETKTGTVDLATLPDRSQTFYRGALRIEWTIKK

VEDGTFKLGVPEEARSSDRSAQQFLEILTSQGLYARGDDHLIFRNQRYDVQENAPVDKKS

TYPFCVRQGVQKSVILCESAEKIATILQLEKKTSPFFPEMNLLDFVRQCSSDDKAMAVLK

GLQVQTTHLKRQQKTFRISGYSNTACKDIHFENRDGEKISVPEYFLKHHAFRTAAGQLPC

VEEKRKPQNNHYPMDCLKIVGGQRILSQKQEPEIVEHLISTARILPLKMADEIGEQLGKH

ILNREAETFLRTFKVNVDRNLLETEATLIKAPQIQYGKNQNGVNPAKTLTTERGQKNAWK

MEDSLTFYRPGVVSDSGEAHRWMFVILNEPNQREHGDCRVFLEKFVARAGTRGIKIQFPH

VEKKRIENSDPWPELQEIGQYAKSNGVKFVMFVYERTGDIRKAMKLLETTFALTTQHVSL

KTISKAAGDKGAFMVLDNLLMKTNEKLGGLNTRVKAEPRVAEWFERGTTMFMGFDVSHPG

LGARNENGVTPTAVGMSFTKNSDLEVVGRLWYQEPREHLIPNMKDHIVEALETFKKHSGK

FPELVVVFRGGVSEGEYEKVQTKEVQEFQDAFQQLKFSRKPILKIVVVQRNSGYRLMPAQ

RNDFGYQKNEALVQNVVPGTCADAEIVDQKRTEFVLVPHQAIQGTAKPSKYVLLHDEAPK

MSKEELSTIAHTLCFMHGIVTSPVSCPSILYQAGDLAKRASCNFKAFLNRKGGNVPIPPV

EEKEKRKEFFDALCEKLKITLDTRFWA

>WAGO-11 (Y49F6A.1) #2 - L596_g17422.t1

MPFPRGKGYHPYRGKSYHRGFGRNNFQYRQTKREEYHSSDSSPTDNQPSTSRSHDRCDDY

KPKDEDDMKLYLSDRKPSPEPPEFQIRINAFPFNIDHAPKEVHTYELIFVMSQKLKKEKE

PANHKLRTNAKLFSAEDIGLGWFDAEEEGQVYHGTDMSCGPTDVVRRQRRKALLFQLFRH

LINQSKEYFPGSKYVYAYDGQRILYSPEVLKMDEGMFMAQLTDLPESVTQLLGPSDQENT

EINAYIRKAEDVIDLHDFGTEARPIHGIEGFLETLTWQHAFEGFEEHIVYGTRYFALNAG

KKLKHCTGFKSIVGFEKRIELLPDYTCSNILLERSLRPVMRMTPKFDLFFDNSTAISLDD

FAVLFFRVEVDDLSATLQKKENLQKLNQVFKNAVVRTIHRDDGRQDTFMIDHLDERNPFE

ITLKEEDEETVADYLYGVYGYKVDRNDRLPCVARKFKNEFAYYPLRTLVLLPNQKVASKF

LPEDMRKSYQEACQNLPSDALNNIFTAMSQLNLSRASEDYNNDEKVIHVNNKYMENFKIT

LESNQLIQMPAQRICEPRIAYRESTHLAQDGAWDYEKAAVFIDAVIKVRRIGLVNTCEDV

GEDDLGEFISKLIGWLRNKTIDLKLTPEDIFWWPDAWNEFKAGKSKKEMLATAEKIIETS

EVKLMFVICSGSADDEIHDIWKLAEVTNELVKVNKKDFVTTQCITPATLSNVLVKSTYQD

EILTNIIMKMNLKLGGTNYVLSKSPSNNEMSIPHIHSSRMFVGIDVLQPEKTQLNGEPTN

NPTVVGISFTDATSKFYLRGTYWYQQSPTVSLCLLQKHFDEALYWYDCHKMDSVPREVFV

YWRDSRIQKNMEEVKMVLEDVICNRAKDVRRDASKLFLIMVDTKPKTRLFTWETQFSGNA

QTQNVQAGTFVRESYRKRQFTMINHKSGAGLAHPVRFTMINDDVNEKDYVEAELEKTTNA

LCFLQNTSTRSTSIPAPLYSAMDLAKRGMKNYETMDAVMREEERDEDRKKREARTPEAWH

RYYKQLVKTHMSVMPIRDSKFWA

>WAGO-11 (Y49F6A.1) #3 - L596_g18655.t1

MSPPRNGSNFNRGNGHKRPPHGHNSQAKRVKWEEHSSDSSPIYNQPSTSRSYDRQPKEEN

DVKPLFSSRERSPRLRDLQIRINAFPFNLDHAPKEVYTYELIFVMSQKLKREKEPENYKI

RTNAKLLEAEDVGLGWFEAEEPGQFYHGTDMSCGPMDVVRRQKRKALLFQLFRHLINQSK

EYFPGSKYVYAYDGQRILYSPEVLKMDEGMFMAQLADLPESVTQFLGLDDQKNTEINAYI

KKSDEVIDLHDFGTESKPIHGIEGFLETLTWQHAFEGFDKHIVYGTRDFALNAGKKLKHC

TGFKSIVGFEKKIELLPDYTSSNMILERRIHPVMRMTPKFDLFFDNSTAISLEDFAVLFF

RVEVENLPATLQKEENLQKLNQVFKNAVVRTNHRDDGRQDTFMIDHLDKRNPFEIIVTEE

DQETVADYLYDVYSYKVDRNDRLPCVARRFRNELAYYPLRTLVLLPNQKVASKFLSEDMR

KSFQEACQNLPSDALNNIFTAMSQLNLSRASEDYNNDEEVTHVNNEYMENFKITLESNQL

IQISANRVHEPQIAYQKSVKPAQDGAWAYEKGAFFVHPKKGVRKIGLVNTCEDVKEDVLG

EFISKLIGWLRNKDIDLKLTKEDIFWWPDAFSDFGVMKSVKEMLAKAEKILETSAVNHMI

VICNGSAEDKTHDVWKLAEVTNGLAKKDRTNFVTTQCITPTTLGSILVKSTYQDEILTSV

IMKMNLKLGGTNYVLTNSNRNYMSVPHITQDRMFVGIAVLQPEKTRLNGDATYNPTVVGL

SYSEGSPNFYLRGTYWYQQSPSVDLSILKKTFAEALYRFDKFHLIPKEIFVFWRSGKFQR

SMEEEKIALQEVIDKRVKDVNAKSPKLFIISVNTKPKTRFFTWETKFSGNAQTQNVQAGT

FVQESFLRREFTMINHKSQAGLAHPVRFTMLNDEIGEQDYAEAEIERTTNALCFLQNNST

RSTAVPAPLYSAMDLAKRGMKNYETMDAVIREEESEEERKKRETRTPEGWQKYYGNLMER

HMPVPIKSSKFWA

>WAGO-2 (F55A12.1) – L596_g16917.t1

MHAHRLQQLTDGINRNLNDEGIARRTPMSPTPSVGRHDKEITLTSNLYELHLAHGKVRVY

HYTIKLSDYTGKTAEVHGDYGRVNLGSKLLLAADTFMKRNGFRDHDFFCDLAGMRLYTIK

PIPRSEGNPRFYVSEATKDGNFAVCRDDGRPFELNIRDVFKEVRIPNLQDATLDAFINTA

INKACLAKSDLYFPIFETCYPKTGAPVARKDGRSLILGTKTSVERVEGRYGSSTTPVVAL

NVSAEWFYMKKNLLDFCKQNIELNEKSVPVDAASFQLLSDSMQGVTVRLICSKNPLLFTI

KSLANKNVSMLKYPLPNIKGTGLIKFLWETYSVRLTYPESFAAEVKPDFHVTDPRPIFYP

VELLEIMPMQRALKGKKSCDGSAVDREKKIQEKVHQMERHVQKAGLGLSMDDAPVEVEAG

VLDLPKIVFADEKVVEVNPSSASWRFGSGECPERFARPAEVKELPWCVMLVSDVPPTKGM

SKKAKFFADLLKKQAAERGLQMKEPMYHPTKQGKVEIERFFNTTAVEFFVFLMAKSLDYH

DFTKILERKYQVITQTVKMENAFDVVEDPNSTKSRKIVEHIVMKMNLKLGGVNSTVKPSR

HLFSHEEVRRLLIGFTLIEGPKITGRDAAAFNRFGGKIPAVVGFSANMGSLEHEFLGDFT

FQYLDHTNIVQDIKKIVATILERFRKTRRGRDPAEVVIYRKLDEFHFPRVIQNEIAPLKN

LLEEKCHGFVSLIYIAMTKTHNIRFFPKGPPPEGDERKPNLVPGTVIDSGAVGGGLKQFF

IASYSASTGTTRPPRFTILENSKEAKIRDLEKLTLELTFAHQTSTRSLGVPAPLVVAKNY

ARRGNVLAKNEAEKISDVDTSVGVETMIDRLPYADAPVLRDKRVTA

>WAGO-5 (ZK1248.7) #1 - L596_g12936.t1

MEGEGTELRPSTVVRQARNAAASSAGAPPAAPGSRAATASVEKSFYSKLVNVPTPPPEEK

KPHGTAGRQLKLRTNVYGLSLPKDVQVFRYSVDASGTLQRNDLRIEFAKRVANDITYLNR

REKCRRVIDQVVAKYSAIFGDRRELFWYDSQSILFSRNQLDISSEGQFVLDQSDIGQNPL

FEGFAHLKMVIRPAQTNFAVSIGDLEAYIQAEVFESDHALQQFLEILTAQYAFNTPLEAM

SFGSRTAYLLNPEKYGFKPADCADVGDGKFLGVGCDKSVRFIEGPGGAGSQRAALVVDLK

KTAFHKDQSLYEKAREILNNRDPKSTDASRLRMQLKGIVVETKHGSRRQEFAVDNVVADT

PATKKFKDLTGQEVTLQQYFQQKYNITLQHPDSPIVLTDRTKKFAAFPMEVCSVVDGQRV

TLAQQTPVQIQKMIRQCAVPPADRQRQILGLVQGLQLNSENKYHKAASVGITPTALQVQA

RLLQNPTIVYGRNSTMKPDEKATWRLARQKPVYLKPAKVDKWAMFVICGGNRSDCVDQDI

LNQFSNMMVQECRARGMTVSDPTGFSFIGASREVVQETLEKAKTEGNQFCFFITNNDVTN

IHQFMKFQERKLSIVTQDMKMSSAFDVVRKGKRQTLENVVNKTNMKNGGVNYSLRFDDPA

FSMEKLLPKDRLVIGLATTHPKPIVGKKEQDEGPHDKKKQMHQQRTGPPVPSVVGVAANA

LTESIEIVGDCLFQQPNREEKIALLQPVIRSLMLQFMKHRGMPPAEIVVYRQGTSEGQFR

DVMELEYKMVKAAALQQGLNPKITFIVVQKMHNVRLMPMDSKAGDKAPEQNVKPGTVVDT

MVTHPKYNEFFLNSHVALQGSARTPRYTVLYDENRLPMDEIEALSHSLAFGHQIVNLTTS

LPAPLYIANRYAERGHNIFIASQEDYTKSKTSFQSPHSTTIEGNLDFGRMMNELSYCNSE

LKDKRVNA

>WAGO-5 (ZK1248.7) #2 - L596_g12936.t1

MEGEGTELRPSTVVRQARNAAASSAGAPPAAPGSRAATASVEKSFYSKLVNVPTPPPEEK

KPHGTAGRQLKLRTNVYGLSLPKDVQVFRYSVDASGTLQRNDLRIEFAKRVANDITYLNR

REKCRRVIDQVVAKYSAIFGDRRELFWYDSQSILFSRNQLDISSEGQFVLDQSDIGQNPL

FEGFAHLKMVIRPAQTNFAVSIGDLEAYIQAEVFESDHALQQFLEILTAQYAFNTPLEAM

SFGSRTAYLLNPEKYGFKPADCADVGDGKFLGVGCDKSVRFIEGPGGAGSQRAALVVDLK

KTAFHKDQSLYEKAREILNNRDPKSTDASRLRMQLKGIVVETKHGSRRQEFAVDNVVADT

PATKKFKDLTGQEVTLQQYFQQKYNITLQHPDSPIVLTDRTKKFAAFPMEVCSVVDGQRV

TLAQQTPVQIQKMIRQCAVPPADRQRQILGLVQGLQLNSENKYHKAASVGITPTALQVQA

RLLQNPTIVYGRNSTMKPDEKATWRLARQKPVYLKPAKVDKWAMFVICGGNRSDCVDQDI

LNQFSNMMVQECRARGMTVSDPTGFSFIGASREVVQETLEKAKTEGNQFCFFITNNDVTN

IHQFMKFQERKLSIVTQDMKMSSAFDVVRKGKRQTLENVVNKTNMKNGGVNYSLRFDDPA

FSMEKLLPKDRLVIGLATTHPKPIVGKKEQDEGPHDKKKQMHQQRTGPPVPSVVGVAANA

LTESIEIVGDCLFQQPNREEKIALLQPVIRSLMLQFMKHRGMPPAEIVVYRQGTSEGQFR

DVMELEYKMVKAAALQQGLNPKITFIVVQKMHNVRLMPMDSKAGDKAPEQNVKPGTVVDT

MVTHPKYNEFFLNSHVALQGSARTPRYTVLYDENRLPMDEIEALSHSLAFGHQIVNLTTS

LPAPLYIANRYAERGHNIFIASQEDYTKSKTSFQSPHSTTIEGNLDFGRMMNELSYCNSE

LKDKRVNA

>WAGO-5 (ZK1248.7) #3 - L596_g17875.t1

MVCKGQATSEIELTTNAYGFLPFLSAKVFQYDVEIVGILSGTGRTVNFTKCSKHDAFRAA

RIEECRDLFEMVKQKYPEVFNAPDSNYFYDNGRRLFTKESLLPPLVSKQEFPLNEFDTVY

LDRDYSRFDKILFSVEKAAEDPMDVKEVLKHVKDSGKSIRLNQFLNVLTSQHALSNPVKF

ATYRIGSAFFINADRYDHNHGVEDLTDDKEIRVGCEKSVKLIAGSSDDGSAIALVDIKRT

AFHKSGGTLLKKAREVLGKWPQPCDANRLKPHFIDLAVYTQHGSKDRRYVIDDVIAETPA

TLKFAWVRGSKELTLVEYFHQAYSIEIKFPRTPLAVAMAKEGRTIYLPLELCFVSPHQRV

TTAQQQATEEMIKRCSIPPLDRQKRIGRIVEAMKISDNPFLQIADTGCKLLSTIPLSVTG

RVIAPPKIRYGHSEIQKSAFLKPAKINSWAIVVLTAQDDPELARGDILSPSVLTKFARLF

RKECKARGMQLPDPFLKEFMKADVDQLGELMRCLAQGDSTECRPPLRFLIFVTNEKLTDL

HHPMKYFERQCGIITQDMKMQTVVDVVLHKKRQILENIVSKANIKNGGLNYSVIIPDVPG

KRPILGSGRLIVGLFTSPTFKWQFEDSPSRPTALGYAANTTPNEGEFIGDCLIQKGRASA

IQTILRRVLAEFKKQRRCDPADVIIYRSCEEGREKKTLEEDLAAVRSILKSSNPMPTLTV

IAVQKRHGLRLMPTAIQRQGEPQSENLKPGTVLDSCLTDPALTEFYLNSHATLQGTARTP

KYTVVHNDVGLSLEEMETLTYALSFSHQIVPLPTSLPSPLYIAGTYAERGVSLYQQDRER

GREGSLESCFTYGSSPGLKHLRITA

>WAGO-5 (ZK1248.7) #4 - L596_g24399.t1

MDVLTEAMSKMLPMNIAPKIVGAQDPYERVVPLTANMFPLHMRAEVPIFMYNVQVFMKVG

FREVNLVKRNTDDFTIIDHKNKCRSAFRFAVRAAPQVFGHPSGLFYDIQAQLYSVRELKD

VLGNDLKKKEEIIVPGEDARKAYDFQDIDLEYLRLVIEPVNGTNPSINLGELVLKQKNFS

DEVPCELLQFLDVACSQHAFLTPTKFTTYPGGFAYFNPTSEEPARELPDAARLHNGVHKS

VKVIEGSCTAGRCGELAVVLDPKKAAFHKPDITVVQKIQEMGFLQMASENVAPHRIPELA

EALKNVFVETRHGKRRSRFAIHSVVAESARTNRFTKDDGQVTVEEHFKKEYDIALKYPHL

PLVVSMPLRKKTPSNGRAPPRLLFFPMEVLFICPNQRVLRNQQSAKQNNEVIKSCAVAPE

HRLKDVIASGQKMRINGPNVHGCLNSAQIQVESEPLKVEGRTLVPPNIDYKGCQVQVDSF

TGKWRNFGRNKPHYLEGGKIGRWGLYVLSKAPSSEEEQLALKFKDKMLVEFQSRGMQIDL

PMFLATVKATPLYLRTIFEKARKERLEFLFFIQDKDLALHNEMKFYERAYEVITQDLRTD

TARAVIEQGKNLSLENIIAKLNVKVGGTNYSVNGPSVPDLFKKGRLYIGLQASTNGPPAA

GAHLPTVVGSAANVTTAPSSFVGDIYFQKFGEMDLQGAMASTTEGYVKRYAAVHGRAPDE

VFIYRSGTANTNIGQMLRDEVPAIRCALKNSGASRARLTLVMVTKQHNVRLMPTNMTLGG

RAIDQNIKPGTVVDQKITHPRFAEFYLNSHQALHGSAKTPKYVVVADDCSNPIQYLERVT

YALSYGHQIVGMPTSLPSPVYIAGKYAERGAALLQTKRNLGGALDVDALAEELAYANSKV

LGFKRINA
